# General Direct Anticancer Effects of Deer Growing Antler Extract in Several Tumour Cell Lines, and Immune System-Mediated Effects in Xenograft Glioblastoma

**DOI:** 10.3390/pharmaceutics16050610

**Published:** 2024-04-30

**Authors:** Alessandra Rossetti, Louis Chonco, Nicolas Alegría, Veronica Zelli, Andrés J. García, Carmen Ramírez-Castillejo, Alessandra Tessitore, Carlos de Cabo, Tomás Landete-Castillejos, Claudio Festuccia

**Affiliations:** 1 Department of Biotechnological and Applied Clinical Sciences, University of L’Aquila, 67100 L’Aquila, Italy; alessandra.rossetti@univaq.it (A.R.); veronica.zelli@univaq.it (V.Z.); alessandra.tessitore@univaq.it (A.T.); claudio.festuccia@univaq.it (C.F.); 2 Instituto de Desarrollo Regional (IDR), University of Castilla-La Mancha (UCLM), 02071 Albacete, Spain; louis.chonco@uclm.es (L.C.); nicolas.alegria.aravena@gmail.com (N.A.); andresjose.garcia@uclm.es (A.J.G.); 3 Instituto de Investigación en Recursos Cinegéticos (IREC; UCLM-CSIC-JCCM), University of Castilla-La Mancha (UCLM), 02071 Albacete, Spain; 4 Escuela Técnica Superior de Ingenieros Agrónomos, Montes y Biotecnología (ETSIAMB), University of Castilla-La Mancha (UCLM), 02071 Albacete, Spain; 5 Cancer Stem Cell Research Group, Department of Biotechnology-Vegetal Biology, Centro de Tecnología Biomédica (CTB), Universidad Politécnica de Madrid, 28040 Madrid, Spain; carmen.ramirez@ctb.upm.es; 6 Research Department, Neuropsychopharmacology Unit, Complejo Hospitalario Universitario de Albacete (CHUA), 02071 Albacete, Spain; carlosd@sescam.jccm.es

**Keywords:** anti-tumour activity, cell proliferation, deer velvet antler, glioblastoma multiforme, immune tolerance

## Abstract

Deer antlers are the fastest growing tissue. Because they are based on proto-oncogenes, to avoid the risk of cancer, antlers evolved strong anticancer mechanisms, and thus their extract (DVA) is effective also against the few human tumours studied so far. We assessed whether DVA is a general anticancer compound by testing the direct effects in cells of different tumours: glioblastoma (GBM; lines U87MG and U251), colorectal (CRC; lines DLD-1, HT-29, SW480, and SW620), breast cancer (BRCA; lines MCF7, SKBR3, and PA00), and leukaemia (THP-1). DVA reduced the viability of tumours but not healthy cells (NHC; lines 293T and HaCaT). Mobility decreased at least for the longest test (72 h). Intraperitoneal/oral 200 mg DVA/kg administration in GBM xenograft mice for 28 d reduced tumour weight by 66.3% and 61.4% respectively, and it also reduced spleen weight (43.8%). In addition, tumours treated with DVA showed symptoms of liquefactive necrosis. Serum cytokines showed DVA up-regulated factors related to tumour fighting and down-regulated those related to inducing immune tolerance to the tumour. DVA shows general anticancer effects in the lines tested and, in GBM mice, also strong indirect effects apparently mediated by the immune system. DVA may contain a future anticancer medicine without secondary effects.

## 1. Introduction

Cancer is one of the most challenging health problems of the XXI century. Worldwide, an estimated 19.3 million new cancer cases and almost 10.0 million cancer deaths occurred in 2020 [1]. The same study showed that lung cancer remained the leading cause of cancer death (18%), followed by colorectal (9.4%), liver (8.3%), stomach (7.7%), and female breast (6.9%) cancers.

New innovative solutions are being developed to address cancer disease. In addition to strategies designed by researchers, often a highly effective solution for solving health problems is to find the model where nature itself has already evolved such a solution. For the case of cancer, nature seems to have evolved anticancer mechanism in the growing deer antler. Antlers are bony cranial appendages that are cast each year and grow (in red deer) in about 4 months [2,3]. They constitute the only case of full regeneration in mammals, reaching weights of more than 13 kg and 116 cm in length [4,5]. This represents an astonishing growth rate in the tips (where they grow) of up to 2.75 cm/d in length [6]. Such fast growth led to unique characteristics with potential medical applications [7], with anticancer mechanisms being one of them. Wang et al. (2019) showed that antlers evolved a speed of growth faster than cancer based on high expression of proto-oncogenes [8]. Indeed, the study found that gene expression profiles between antlers and that of osteosarcoma are more correlated (r = 0.67 to 0.78) than between antlers and normal growth in bone tissues (r = 0.33 to 0.47). As a result, the study postulated that deer evolved several tumour suppression genes and mechanisms to reduce the high risk of developing cancer.

This is most likely the reason why the extract of the growing antler (called deer velvet antler, henceforth indicated as DVA) shows anticancer properties both in an array of human and mouse tumours. Fan et al. (1998) published the first evidence of the anti-tumour activity of DVA in mice inoculated with sarcoma 180 cells [9]. They observed that DVA-treated mice intraperitoneally significantly prolonged the life of the cancer-bearing mice from 15 to 20 days. These results were confirmed later in the same animal model with DVA extracted from the tip (henceforth DVA-TIP) [10]. Furthermore, DVA showed proliferation reductions in cell cultures of human prostate cancer similar to those of chemotherapy [11], whereas in xenograft mice, DVA achieved a 65% tumour volume reduction, again similar to that of cisplatin chemotherapy [12]. In malignant glioblastoma (GBM), the most prevalent and aggressive malignant brain tumour, DVA showed a reduced proliferation (37.5%) and colony-formation capacity (84%), inhibited migration (39%), induced changes in cell cycle progression, and promoted apoptosis, whereas it did not affect non-cancerous human (NCH) cell lines [13]. Other studies have shown that DVA or its proteins exert anticancer effects in human breast cancer (BRCA) [14,15] and mouse tumours such as colorectal cancer (CRC) [16] or sarcoma [17]. Since the growing antlers have a variety of tissues (among others, skin, cartilage, bone, blood vessels, and nerves), Landete-Castillejos et al. (2022) suggested that deer antlers may show a general anticancer activity, particularly in the tips, the growing section [18]. Furthermore, because these mechanisms are acting in a live mammal, this explains why it has no negative secondary effects in other tissues and cells reproducing according to healthy physiological processes, and no such effect is expected in future studies. This makes for a very promising line of research.

How could such anticancer effects be achieved? The limited research so far shows direct effects on tumour cells, and others derived from DVA potentiating the immune system, as shown in the paper by Cao et al. [17]. In this study, DVA was given as preventive anticancer treatment to mice for 15 days before injecting murine sarcoma 180 (S180) cells. The weight of the sarcoma was smaller the higher the velvet antler protein (VA-pro) dose (one of the components of DVA): ranging from 25% to 55% tumour weight reduction from the lowest to highest dose of VA-pro. The reduction in tumour weight seems to be explained, among other mechanisms, by the results in the study showing that tumour cell proliferation was arrested in phase S. The apoptosis test (Annexin V-FITC/PI) showed that tumour apoptotic cells increased from 6% (control) to 76% (highest DVA dose), whereas normal sarcoma cells went down from 91% (control) to 19%. All of this would explain the direct anticancer effects of DVA shown in vitro in other studies (although in vivo, apoptosis can be induced by cytotoxic T cells; see below).

However, this study and others showed also immunomodulatory properties [19] and indirect anticancer effects mediated by the mouse immune system [20]. To understand the role of immune system in cancer, it is important to discern between effects aiming at killing the tumour cells, and the opposite effects induced by the tumour to create a immunosuppressive microenvironment in the tumour to avoid precisely immune attacks on cancer cells [21,22]. The tumour is infiltrated by cytotoxic CD8^+^ T cells to kill tumour cells, a response that is often mediated by CD4^+^ T cells, which are trained for the immune response in the thymus [23,24]. Both Li et al. [15] and Cao et al. [17] showed that DVA could increase, in mice, the number of CD4^+^ and CD8^+^ T lymphocytes, in addition to IL-2 and other interleukins involved in the immune response against cancer. Equally, the size of thymus increased with increasing dose of DVA, likely paralleling the rise in lymphocytes involved in anticancer response [17]. To successfully develop, tumours must escape immune surveillance by developing an immunosuppressive microenvironment that induces immune tolerance [20]. This tumour microenvironment contains various immunosuppressive cells, including tumour-associated macrophages (TAMs), myeloid-derived suppressor cells (MDSCs), and tumour-associated neutrophils (TANs), which contribute to immune tolerance and tumour progression [24,25,26].

From these, TAMs account for most of the proportion, particularly M2 type which is dominant in most solid tumours [27,28]. In fact, M2 protects the tumour cells from chemotherapy reducing apoptosis, whereas another one, M1, is pro-inflammatory and cytotoxic, increasing the apoptosis normally produced by chemotherapy alone [29]. Surprisingly, these types can be polarised from one type to another [22,28], so that one way of fighting cancer is to re-polarise M2 into M1 [30].

Another immune organ involved in fighting cancer, the spleen, was also influenced in mice with tumours treated with DVA [17]. Previous studies have demonstrated that the spleen is an important site of extramedullary haematopoiesis, and, in tumour-bearing mice, the spleen generates immunosuppressive myeloid cells [31,32]. These cells also induce the tumour immune tolerance mentioned above [21,33]. These myeloid cells promote tumour progression by regulating the anti-tumour immune activity of T lymphocytes, natural killer T (NKT) cells, natural killer (NK) cells, dendritic cells (DCs), and various other cell types [34]. The study by Cao et al. [17] showed that the greater the dose of DVA, the smaller the spleen and the closer its weight to that of the healthy mice (whereas the greatest spleen was that of control mice with tumour). Thus, spleen size may be related to the production of the immunosuppressive myeloid cells, thus promoting tumour growth, but also monocytes that infiltrate the tumour differentiate into macrophages (called M0) and then polarise into M2 or M1 [27].

Thus, the aims of this study were to assess the direct anticancer effects of DVA in several tumour cell lines as compared with healthy cells and examine in detail the modulation of these effects plus its potential mechanisms in the immune system in GBM xenograft mice. In addition, we aimed to assess whether DVA can modulate the M1/M2 effect on GBM (as an example for other cancer cells). Thus, the specific aims were (1) to assess the anticancer effects of DVA in vitro in cell cultures of GBM (cell lines U87 and U251), CRC (lines DLD1, HT29, SW480, and SW620), and BRCA (lines PA00, SKBR3, and MCF7) and, additionally, if these effects are also exerted in tumours such as THP-1 leukaemia, which affect the immune cells; (2) to assess the validity of these direct effects in vivo as assessed in the weight of the human GBM xenograft tumour in mice and its histopathology; (3) to assess the potential mechanisms of these effects modulated by the immune system via assessment of the weight of the spleen and cytokines; and, in particular, (4) whether DVA can affect the immune microenvironment of the tumour by assessing the viability of GBM in vitro with supernatant of M1 and M2 with or without DVA.

## 2. Materials and Methods

### 2.1. Antler Samples

Antlers were sampled from 12 adult males of red deer which were hunted for other purposes (summer selective shooting to reduce population density) in a deer private game state in Ciudad Real province (38°53′ N, 4°17′ E). Males were chosen among those who had antlers in a growth stage similar to farm animals at 60 days of age (from our experience in the experimental deer of the UCLM and guidelines from Deer Industry New Zealand). Immediately after death, antlers were cut off with a mechanical saw and then kept refrigerated until they were stored frozen at −20 °C until they could be freeze-dried. Then, they were divided into portions according to the distance from the extreme of each antler (main beam, bez tine and brow tine were developed with 60 days of age). The tip is considered, in every antler, to be 2.5 cm section at the top. Then, middle portions are considered to be 5 cm below the tip section, obtaining 2 or 3 mid-sections depending on the deer age. Only the first mid-section (2.5–7.5 cm from the top) was used in this study. The middle portion used in this study has a functional difference from the rest of the antler: according to Muir et al. [35], the transition from mineralised cartilage to trabecular bone occurs in a discrete band located from 5.0 to 7.5 cm below the antler tip (i.e., our mid-section). Each portion was separated, and powder was obtained through a grinding process in ball mills until particles were less than 0.18 mm. The weight loss after freeze-drying (i.e., corresponding to the percentage of water in the fresh sample) was 78.4 ± 2.5 (%) and 74.4 ± 2.7 (%), respectively, for the tip and first 5 cm mid-section of the main beam, and 77.9 ± 1.2 (%) and 71.6 ± 1.5 (%) for the tip and first 5 cm mid-sections of the first and second tine of the antler.

### 2.2. Production of DVA Extract and Protein Quantification

The DVA powder (1 g) was weighed and soaked with 10 mL distilled water. The liquid mixture was incubated at 4 °C overnight, with continuous stirring, and then centrifuged at 2700× *g* for 20 min. The supernatant was freeze-dried and dissolved into 2 mL Phosphate-Buffered Saline (PBS). Samples were frozen at −80 °C; and to carry out any further assay, DVA samples were thawed, passed through a 0.22 μm filter, and centrifuged at 5600× *g* for 3 min. Protein concentration in DVA extracts for further assays was determined by the BCA Protein Assay Kit (Sigma-Aldrich, St. Louis, MO, USA).

### 2.3. Cell Cultures and Cell Culture Materials

U87MG, U251, THP-1, CRC (DLD-1, HT-29, SW480, and SW620), and BRCA cell lines (MCF7 and SKBR3) were purchased from American Type Culture Collection (ATCC, Manassas, VA, USA). PA00 cell line was purchased from a patient luminal B-subtype tumour [36]. NCH cell lines are immortalised human cell lines from kidney (293T) or keratinocytes (HaCaT). Cells lines were cultured in DMEM (High Glucose, Dominique Dutscher, Bernolsheim, France) supplemented with 10% fetal bovine serum (FBS, PAN Biotech, Aidenbach, Germany), 2 mM L-glutamine (PAN Biotech, Germany), and 1% penicillin/streptomycin (Corning, New York, NY, USA) at 37 °C with 5% CO_2_ in a humidified incubator (Series II water Jacker, Thermo Scientific, Waltham, MA, USA).

### 2.4. Cell Viability Assay

Cells were seeded on 96-well plates (Deltalab S.L, Barcelona, Spain) at a final concentration of 20,000 cells/well and were left in the incubator for 24 h at 37 °C with 5% CO_2_. Then, DVA was added in increasing concentrations—0.1, 0.5, and 1 mg/mL of protein extract—and were left to incubate for 72 h. The MTT (3-(4,5-Dimethyl-2-thiazolyl)-2,5-diphenyl-2H-tetrazolium bromide) assay was used to measure cellular metabolic activity as an indicator of cell viability, proliferation, and cytotoxicity. The reactive was added to 0.5 mg/mL MTT solution and was incubated for 4 h at 37 °C. Then, it was emptied, and DMSO was added (Labbox Labware, S.L., Barcelona, Spain). The detection of absorbance was read at 546 nm, using the spectrophotometer BIOBASE-EL10A (Biobase, Jinan, China). The amount of colour produced is directly proportional to the number of viable cells. The cell viability was calculated using the following equation: cell viability (%) = (mean absorbance of the sample)/(mean absorbance of the control) × 100.

### 2.5. Scratch Assay

GBM, CRC, and BRCA cells was exposed to DVA extract (1 mg/mL). Rates of wound healing over different time intervals were evaluated to 30 min with Ezscope live cell imaging system (Blue-Ray Biotech Corp, Xindian Disctrict, Taiwan). The cells were left to incubate for 72 h. For the analysis, we used imageJ version 1.54f with Wound_healing_size_tool plugin and GraphPrism 8 statistical analysis.

### 2.6. M1/M2 Macrophages

THP1 plated were differentiated with PMA (Phorbol 12-myristate 13-acetate), with a concentration of 100 ng/mL, and activated to M0 macrophages. Then, LPS (Lipopolysaccharides) was used at a concentration of 100 ng/mL to activate M0 to M1, and IL4 and IL13 were used to activate M0 to M2, using a concentration of 20 ng/mL for both factors. The, 24 h later, DVA Tip was added (1 mg/mL), and the medium was used after 24 h from the last treatment. THP1 cells (in suspension) were used as CTRL and treated with DVA Tip at 1 mg/mL. THP1 treated with PMA + LPS was used for CTRL of M1 macrophages and for those treated with Tip. M0 U87 were set up with similar conditions. GBM cells were cultured with both DMEM and RPMI because THP1s have RPMI (Roswell Park Memorial Institute 1640) as a culture medium. Data were evaluated with the use of Cell Counting Kit 8 (Dojindo, Kumamoto, Japan), and values were obtained through TECAN Sunrise reader (Life Sciences, Tecan Trading AG, Männedorf, Switzerland).

### 2.7. Cytokine Array

Mouse Cytokine Antibody Array C3 was used for evaluating differentially expressed cytokines in sera from DVA-treated mice. Two technical and two biological replicates were performed. The protocol was carried out as suggested by its producer (RayBiotech Life, Inc. Peachtree Corners, GA USA). RayBio^®^ Analysis Software (https://www.raybiotech.com/tools/array-analysis-tool, accessed on 25 April 2024) was used to analyse data. A list of differentially expressed proteins was analysed by IPA. Proteins with significant differential expression levels, less than 0.7 and more than 1.5 compared to untreated cells, were considered.

### 2.8. Subcutaneous Xenograft Model

After 1 week of quarantine, female CD1-nu/nu mice at 6 weeks of age (purchased from Charles River, Milan, Italy) that were followed under the guidelines established by our institution received subcutaneous flank injections (2 each) of 1 × 10^6^ U87MG cells. After the tumour was established and when it reached 80 mm^3^ in volume, the mice were randomised into the following groups (7 mice per group): control (no treatment), oral administration (oral) 200 mg DVA/kg for 28 consecutive days, and intra-peritoneal (IP) injections of 200 mg DVA/kg for 28 consecutive days.

In order to monitor the toxicity of the treatment, the body weights of the mice were recorded twice a week. Tumour mass growth was evaluated twice a week by measuring the diameters of the subcutaneous tumours with a Vernier calliper. Tumour volumes were calculated using the following formula: tumour volume (mm^3^) = 4/3π(r1 × r2 × r3) [37]. At the end point of the experiment (35 days after the start of treatment), we sacrificed the animals with carbon dioxide inhalation. The tumour masses were collected, weighed, and fixed in paraformaldehyde for immunohistochemical analysis.

Statistical analysis to assess effect of oral/intraperitoneal administration of DVA on tumour or spleen weight was analysed using a general linear model with type of administration as categorical variable (with the statistical package SPSS). In addition, spleen weight was subjected to a second GLM using both categorical variables and tumour weight as covariable to assess if covariable absorbed the variability of administration type and increased R^2^ to explain a greater percentage of variability. Both GLMs give complementary information, as indicated in the results and discussion.

### 2.9. Functional Enrichment Analysis

Gene Ontology (GO) and pathway-based analyses of the differentially expressed genes coding for the differentially expressed proteins, as identified by antibody array, were performed using the Database for Annotation, Visualization and Integrated Discovery (DAVID) (https://david.ncifcrf.gov/; accessed on 20 June 2023) in order to assess the biological relevance of up/down-regulated genes within the two groups. GO analysis was mainly performed based on biological process (BP) and molecular function (MF), while pathway analysis was carried out by using KEGG, Reactome, and Biocarta databases. A Benjamini–Hochberg-adjusted *p*-value < 0.05 was used to filter statistically significant terms. The different plots were created using IPA free software R version 4.3.1 (www.r-project.org, accessed on 20 June 2023).

## 3. Results

### 3.1. Generality of DVA Anticancer Effects: Direct Effects on Glioblastoma, Leukaemia, Colorectal and Breast Cancer Cell Lines vs. Non-Cancerous Cell Lines

#### 3.1.1. Glioblastoma

**Figure 1:**
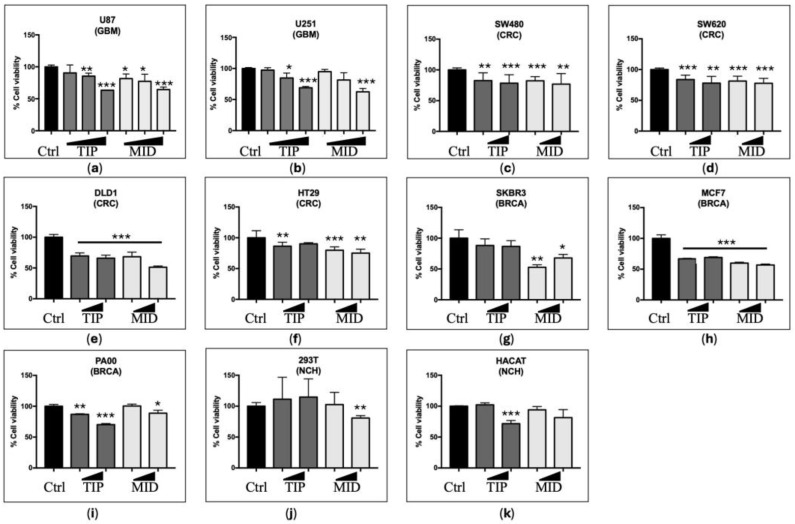
Viability assay for glioblastoma (GBM), colorectal cancer (CRC), breast cancer (BRCA), and non-cancerous human (NCH) cell lines. (**a**) U87 (GBM) cell line has a neuronal-like phenotype with a high proliferative capacity, while (**b**) U251 (GBM) cell line has a mesenchymal-like phenotype with a lower proliferative activity. From left to right DVA doses after 72 h increase from 0.1, 0.5 to 1 mg/mL, whereas, in the rest, it shows only 0.5 mg/mL and 1 mg/mL (to strengthen validity of results in CRC lines, whilst saving DVA extract, tests at 0.5 mg/mL were repeated in 3 experiments). Dark grey colour is used for tip of antler or tine DVA (first 2.5 cm), whereas light grey is used for mid section DVA (next 5 cm). (**c**) SW480 (CRC) cell line is sensitive to chemotherapy and derive from primary tumour. Cell lines (**d**) SW620 (CRC derived from metastasis), (**e**) DLD1 (CRC), and (**f**) HT29 (CRC) are lines of fast-growing and relapsing tumours, in addition to being multi-resistant to chemotherapy. In contrast, (**g**) SKBR3 (BRCA) and (**i**) PA00 (BRCA) are a fast-growing, chemotherapy-sensitive lines, whereas (**h**) MCF7 (BRCA) grows more slowly and is chemotherapy resistant. NCH cell lines (**j**) 293T and (**k**) HaCaT are, respectively, human embryonic kidney and human keratinocyte cells. Bars show DVA cytotoxicity on cancer or control cell lines, whereas asterisks show significant differences with the control (black). Error lines show SD. The probability indicated by *, **, and *** corresponds to a *t*-test at levels *p* < 0.05, *p* < 0.01, and *p* < 0.001 against control (black).

Our group demonstrated, in 2021, the anti-tumour effect of DVA in T98G TMZ-resistant and A172 TMZ-sensitive GBM cell line. We confirmed this activity in another two TMZ-sensitive GBM cell lines with high and low proliferative capacity (U87 and U251, respectively). Figure 1 shows the effect of increasing concentrations of DVA from 0.1 and 0.5 up to 1 mg/mL on the GBM cell lines (a) U87 and (b) U251. Compared with the control (100%), the antler extract reduced, in a dose-dependent way, the viability of both cell lines, ranging from 31 to 38% at 1 mg/mL. Contrary to predictions, the effect was similar both when the DVA extract was prepared from the tip (predicted to have the greatest anti-tumour effect as it is the fastest growing section) or when it was from the middle part (the mineralizing section). Thus, at 1 mg/mL, the DVA-TIP reduced the viability to 63.4 ± 0.4% and 69 ± 4% in U87 and U251, respectively, whereas DVA-MID reduced the viability to 64 ± 4 and 63 ± 5% in these cell lines.

**Figure 2:**
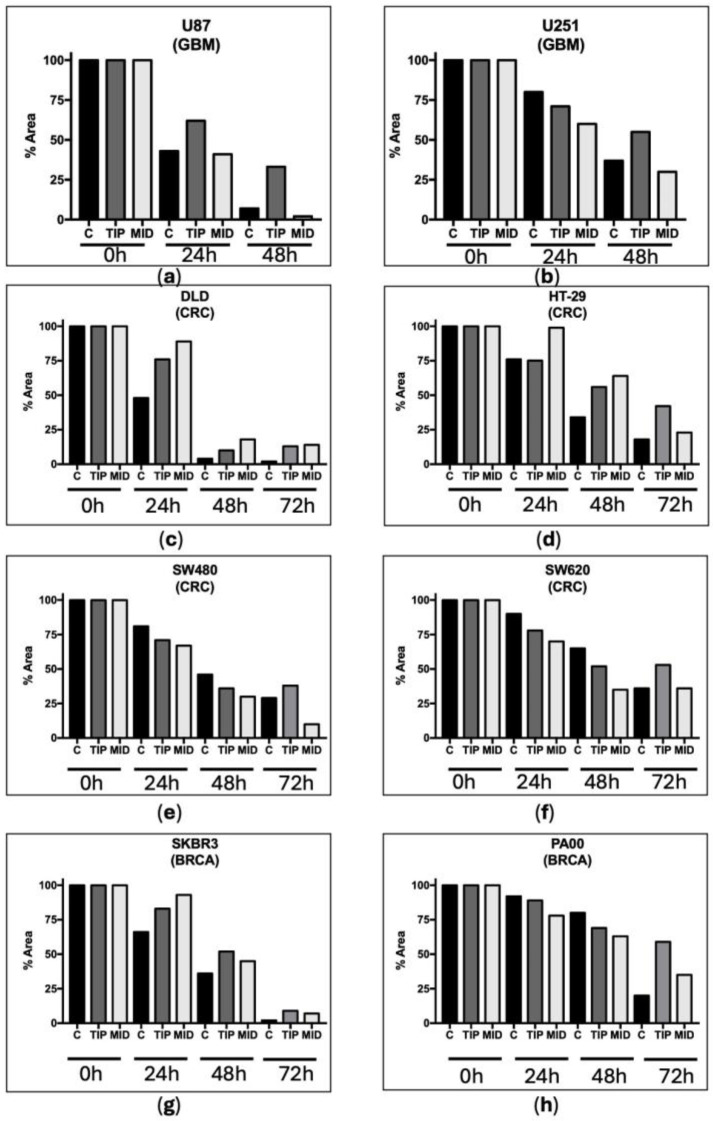
Scratch test showing how DVA affects cell mobility. Bars show how the percentage of surface void of GBM cell lines (**a**) U87 and (**b**) U251 changes from 100% (void) to 0% (fully colonised) after a central line of a plate is scratched to remove all the cells and treated with DVA at 1 mg/mL. The U87 cell line has a neuronal-like phenotype with a high proliferative capacity, while the U251 cell line has a mesenchymal-like phenotype with a lower proliferative activity. Dark grey colour is used for tip of antler or tine DVA (first 2.5 cm), whereas light grey is used for mid section DVA (next 5 cm). Lower graphs show the same test at 1 mg/mL in the colorectal lines tested in the previous assay: lines (**f**) SW620 (derived from metastasis), (**c**) DLD1, and (**d**) HT29 are lines of fast-growing and relapsing tumours, in addition to being multi-resistant to chemotherapy. For breast cancer, the bottom line shows the results for line (**h**) PA00 and (**g**) SKBR3, both fast-growing, chemotherapy-sensitive lines. Time 0 h shows the 100% surface removed from cells. The rest of the bars show how the void space is reduced by colonisation of cells after subjected them either to no treatment (**c**) or DVA-TIP or DVA-MID once at 24, 48, or 72 h. The advance of cell proliferation was measured on the images using NIH ImageJ software version 1.54f. Higher bars at any given time show slower mobility from unscratched areas (estimate of lower metastasis ability). In general, DVA-TIP reduced the mobility of tumour cells as compared to control; however, in lines (**e**) SW480 and SW620, the effect is only found at 72 h.

Regarding the scratch test to assess mobility (estimate of metastasis capacity), the DVA also reduced the mobility of both GBM cell lines with high (U87) and low (U251) proliferation capacity. In contrast to the previous test, the scratch test showed a greater capacity for reducing mobility in DVA-TIP as compared to those of the DVA-MID, which seem to have little effect (i.e., higher bars or void space in the former compared to the latter in Figure 2): thus, cultures from (a) U87 treated with DVA-TIP at 24 h showed a 62% space void compared to 41% with DVA-MID (untreated U87 covered 43%). At 48 h, the difference was even greater (33% for DVA-TIP compared to 2% from DVA-MID, which is actually lower than 7% from untreated U87). The effect was less intense for the slow proliferation line, (b) U251 (71% space void from DVA-TIP compared to 60% from DVA-MID at 24 h, surprisingly the untreated cells moved more slowly and showed 80% void space). These values seem to be rather similar, and only at 48 h did U251 show a clearer reduction in mobility for DVA-TIP: 55% void space vs. 30% and 37% of mid DVA and untreated cells, respectively).

#### 3.1.2. Other Cancer and Non-Cancer Cell Lines

To test the hypothesis that DVA has wide (possibly general) anticancer effects in tumours different from glioblastoma, whilst not affecting NCH cells, we tested it in several lines of colorectal, breast and leukaemia cancer cells.

In general, as shown in the middle and lower graphs of Figure 1, DVA reduced the viability of tumour lines by 21% at 0.5 mg/mL, and 25–29% at 1 mg/mL, whereas it had no general significant trend in NCH cell lines from kidney (293T) or keratinocytes (HaCaT). In a greater detail, colorectal cancer lines multi-resistant to chemotherapy of fast growth and relapsing tumours showed a range of viability reduction greater in (e) DLD1 34–49% (at 1 mg/mL), whereas the effect was somewhat smaller in lines (d) SW620 (derived from metastasis) and (f) HT29 (22 and 17% reduction at 1 mg/mL). The chemotherapy-sensitive line (c) SW480 (derived from primary tumour) showed also a 22% reduction at 1 mg/mL. For breast cancer, the more slow-growth, chemotherapy-resistant line (h) MCF7 showed the greatest reduction in viability (31–43% reduction at 1 mg/mL, depending on antler section of DVA), whereas the DVA reduction in viability was smaller in the fast-growing, chemotherapy-sensitive (i) PA00 and (g) SKBR3 lines (11–32% reduction at 1 mg/mL, depending on antler section of DVA). DVA produced no significant general reduction in viability in NCH cell lines (j) 293T and (k) HaCaT, except DVA from mid-section (but not tip) at 1 mg/mL (but not at 0.5 mg/mL) for 293T, and tip (but not mid) section at 1 mg/mL (but not at 0.5 mg/mL) HaCaT.

We also performed scratch tests in the colorectal and breast cancer lines, as with the GBM line, to assess mobility (Figure 2). For colorectal lines (c) DLD1 and (d) HT29, DVA from both sections showed a slowing effect in all times as compared to control. For colorectal lines (e) SW480 and (f) SW620, the slowing effect was consistently found at 72 h when DVA was extracted from the tip. For breast cancer cells, the slowing down effect (reduced mobility) exerted by DVA was clear at 24, 48, and 72 h in (g) SKBR3. In contrast, for line (h) PA00, the effect was only clear in the maximum period examined (72 h).

**Figure 3:**
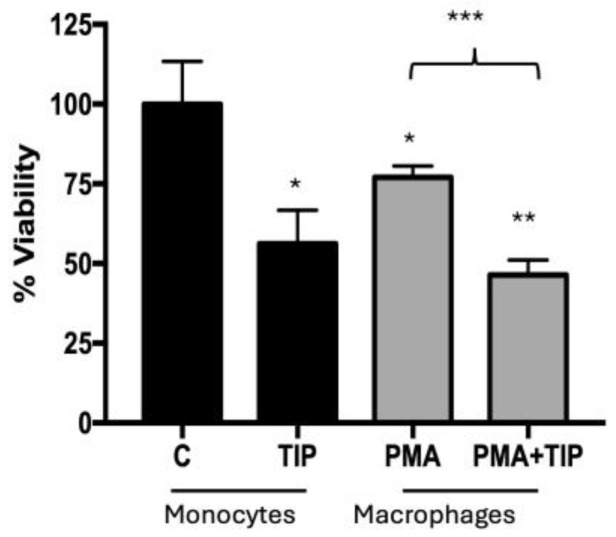
Monocyte/Macrophage viability assay. Viability reduction of leukaemia cell cultures (THP-1) after treatment with DVA-TIP at 24 h. The two bars at the left show the control (C) viability of monocytes (label at the base), and the percentage of viability reduced by applying DVA-TIP at a concentration of 1 mg/mL. The two bars at the right show, respectively, the percentage of leukaemia monocytes differentiated and activated into macrophages after administration of PMA (M0, differentiated-THP-1 macrophages), and the percentage of the latter surviving after the treatment with DVA-TIP. Error lines show SD. The probability indicated by *, **, and *** corresponds to a *t*-test at levels *p* < 0.05, *p* < 0.01, and *p* < 0.001. Asterisks on top of bars show differences with control monocytes (C), whereas those above brackets indicate the significance of differences between the bars at both ends of the bracket.

Because the effects of DVA are exerted through the immune system, we examined also if DVA (from the tip, to assess the most effective extract) reduced the viability of tumour cells of the immune system: THP-1 leukaemia monocytes and their macrophages M1 counterparts differentiated by PMA + LPS (Figure 3). The results showed a substantial reduction in viability of around 40% for both monocytes THP-1 (44%) and macrophages (40%).

**Figure 4:**
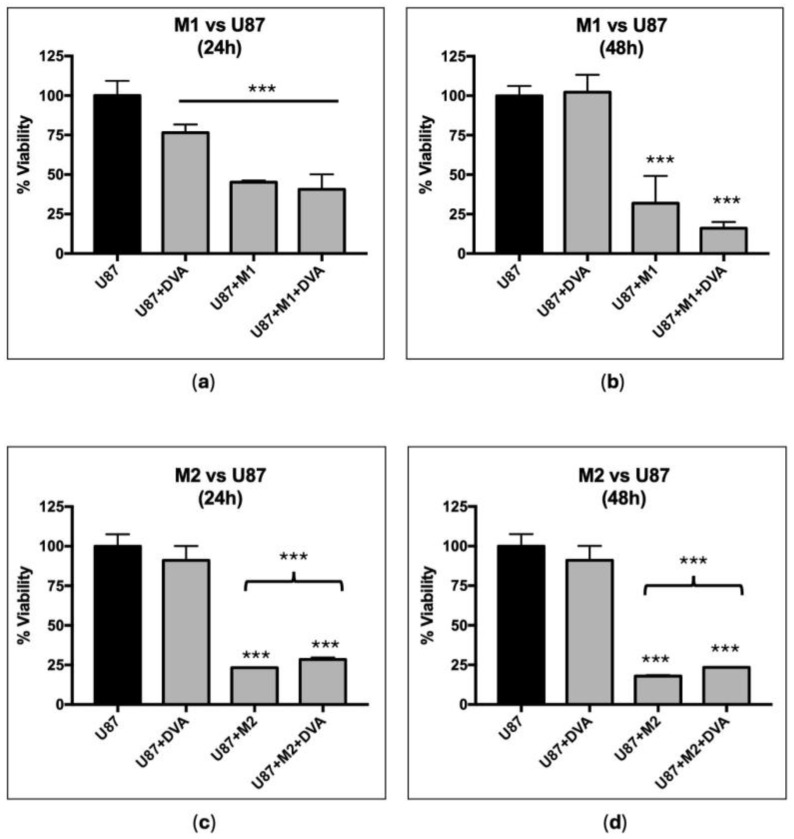
Modulation of macrophages M1 (pro-inflammatory, anticancer, top) and M2 (tolerant and protective of tumour, bottom) effect by DVA in assays with glioblastoma. The bars show the viability of GBM line U87 alone (control), treated with DVA-TIP (1 mg/mL), treated with supernatant of macrophage M1 or M2, and the macrophage supernatant with DVA. The reduction in viability of adding the culture media (supernatant) of macrophages M1 or M2 could be produced by some factor released by M1 or M2, or by a reduction in nutrients caused by macrophage growth. However, as the medium is more than enough to nurture cells for 72 h, the reduction in viability of U87 must be produced by some factor released by M1 or M2. It was expected than M2, inducing tolerance to tumour growth, may counteract the viability reduction of DVA. (**a**,**b**) M1 and (**c**,**d**) M2 are differentiated from THP-1 after treatment with PMA towards M0 and then polarised towards M1 (LPS) or M2 (IL4 and IL13). The graphic on the left shows the effects at 24 h, whereas the one on the right is at 48 h. Data are shown as mean value ± SD. The brackets show the probability indicated of a *t*-test comparing U87 (left end of bracket) with treatment at the other end of the bracket (*p* < 0.001 in all cases). Despite the further reduction in viability of adding DVA-TIP to M1 supernatant at 48 h (compared to M1 supernatant alone: bar next to the left), the test between them did not achieve significance. Error lines show SD. The probability indicated by *** corresponds to a *t*-test at levels *p* < 0.001. Asterisks on top of bars show differences with control (C), whereas those above brackets indicate the significance of differences between the bars at both ends of the bracket.

DVA shows an unclear trend to affect macrophages M1 and M2 (tumour-associated macrophages) immune microenvironment. Figure 4 shows the viability of the different treatments compared to glioblastoma line U87 as control. In all cases, the addition of the supernatant of differentiated M1 or M2 macrophages to the U87 reduced the viability further than the use of DVA alone. As the medium is more than enough to nurture cells for 72 h, the reduction in viability of U87 must be produced by some factor released by M1 or M2 during their growth, thus affecting the viability more than DVA can. The addition of DVA to the supernatant of M1 or M2 did not reduce the viability of U87 further (only a non-significant trend from 40.6% to 45.1% in M1 at 48 h, whereas a significant slight increase in viability was found for M2 both in 24 and 48 h).

### 3.2. DVA Antitumoral Effect in Mice with Xenograft Glioblastoma

#### 3.2.1. Effects at Macroscopic Level

**Figure 5:**
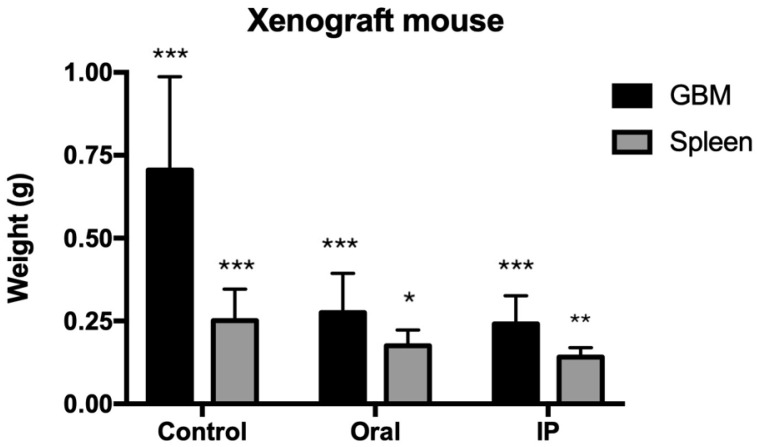
Xenograft human glioblastoma growing in CD1-nu/nu mice treated with DVA-TIP. When the U87MG tumour reached 80 mm3 in volume, mice were randomly assigned 3 treatments: control as non-treated mice (C), oral administration of 200 mg DVA/kg live weight and d^−1^ for 28 consecutive days (Oral), and intraperitoneal injections of the same amount for the same period (IP). Vertical axis shows weight in grams. GBM (black colour) is the tumour weight for each treatment (control, oral, or IP), whereas spleen is the weight of this organ for each treatment. A GLM for tumour weight was GBM tumour weight = 0.706 ± 0.069 *** −0.430 ± 0.098 *** (Oral) −0.464 ± 0.098 *** (IP); R^2^ = 60.6%. Intercept corresponds to the weight of control tumours, and each treatment reduced it, as shown by the coefficients oral or IP (asterisks show the significance level of the coefficient). For Spleen weight, the GLM obtained was spleen weight = 0.251 ± 0.024 *** −0.076 ± 0.024 * (Oral) −0.110 ± 0.024 ** (IP); R^2^ = 38.3%. Intercept corresponds to the weight of spleen in control mice, and coefficients are interpreted as for tumour weight. In both models the superscripts *, **, and *** show the significance level of the coefficients at probability levels *p* < 0.05, *p* < 0.01, and *p* < 0.001.

One of the most interesting results come from the experiment inserting human glioblastoma U87MG on immunocompromised mice. Figure 5 shows the size of the xenograft tumour after 28 days of treatment with DVA-TIP at 200 mg/kg live weight. The figure shows the weights of the tumour for control (untreated mice) and oral/intraperitoneal treatments. The GLM in the legend shows how the weight of the untreated mice (intercept) was reduced by each treatment. In contrast to expected prediction, oral administration had a strong effect (61.4% reduction), despite the fact that the extract was digested. The injected extract had a somewhat greater effect (66.3% reduction; 4.9 percent units more). The variability explained was rather high for an experiment with such reduced sample and considering the variability in immune responses in live mice: 60.6%.

It is also interesting to note the effect on the spleen (dark grey bars in Figure 5). In the spleen, the DVA mimicked the effects it had on the tumour weight. As shown in the GLM of spleen weight indicated in the legend of Figure 5, the oral administration of DVA resulted in a spleen 30.3% lighter than that of untreated mice, whereas the intraperitoneal administration reduced further the spleen by 43.8% compared to that of control mice. Here, IP treatment reduced about forty percent further the weight of the spleen. An interesting result was that, if the weight of the tumour was included in the model, then the treatment became non-significant as tumour weight absorbed its variability and likely that of natural variation in tumour weight, as the R^2^ increased from the previous 38.3% to 52.0% (model spleen weight = 0.14 ± 0.06 [intercept] + 0.16 ± 0.07 [tumour weight]). This suggests that the effect of the treatment in the first model was exerted through reductions in the spleen weight by each type of DVA administration.

#### 3.2.2. Effects at Microscopic Level

**Figure 6:**
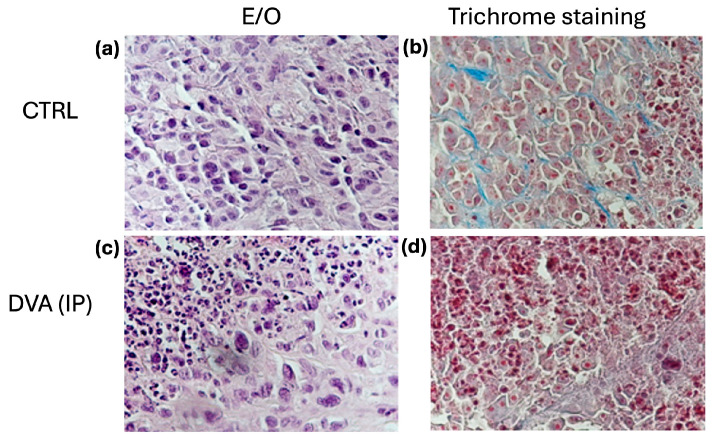
Histochemistry micrographs of glioblastoma treated with DVA intraperitoneally (DVA (IP)) or untreated (CTRL). (**a**) Shows a haematoxylin and eosin stain as a basic stain, whereas (**b**) shows a trichrome stain to see the reaction of the tumour parenchyma (cancer cells) and the stroma (supportive tissue of the tumour) to the treatment. (**a**) Shows that tumours collected from untreated control mice consist of compact cell clusters with little fibrous stroma, and the presence of inflammatory cells being mainly monocytes and neutrophilic granulocytes. In contrast, the tumours collected from mice treated with DVA (**c**), which are much smaller in size, have a different consistency with harder fibrotic portions that are coloured blue/light blue with trichrome staining and hyaline portions that are very soft to the touch with evidence of necrosis, especially of the colliquative/liquefactive necrosis type. This is likely evidence of the direct effects of DVA producing cell death on GBM tumour. (**d**) In treated tumours, necrosis is also abundant with red blood cells due to a weak perivascular matrix, fragile vessels, and frequent ruptures, indicating an additional effect of DVA on tumour angiogenesis. As the tumour reduction was similar, tumours of oral treatments were not examined. Original magnification: 40× corresponding to 1 cm = 200 micron.

The exam of histochemistry slides of glioblastoma treated with DVA intraperitoneally as compared with untreated control showed further effects of DVA (Figure 6). The aspect of control tumours, either in haematoxylin and eosin stain (as a basic stain) or in a trichrome stain, shows that tumours collected from untreated control mice consist of compact cell clusters with little fibrous stroma (supportive tissue of the tumour) and the presence of inflammatory cells, which are mostly monocytes and neutrophilic granulocytes. In contrast to the untreated tumours, DVA produced in the smaller tumours a different consistency with harder fibrotic portions (coloured blue/light blue with trichrome staining) and hyaline portions that are very soft to the touch. This is evidence of colliquative/liquefactive necrosis. In the treated tumours, necrosis is also abundant with red blood cells due to a weak perivascular matrix, fragile vessels, and frequent ruptures. Overall, this evidence shows likely direct effects of DVA producing cell death on GBM tumours, as well as indirect effects on tumour angiogenesis.

#### 3.2.3. DVA Effects on Serum Proteins’ Expression and Related Coding Genes

**Figure 7:**
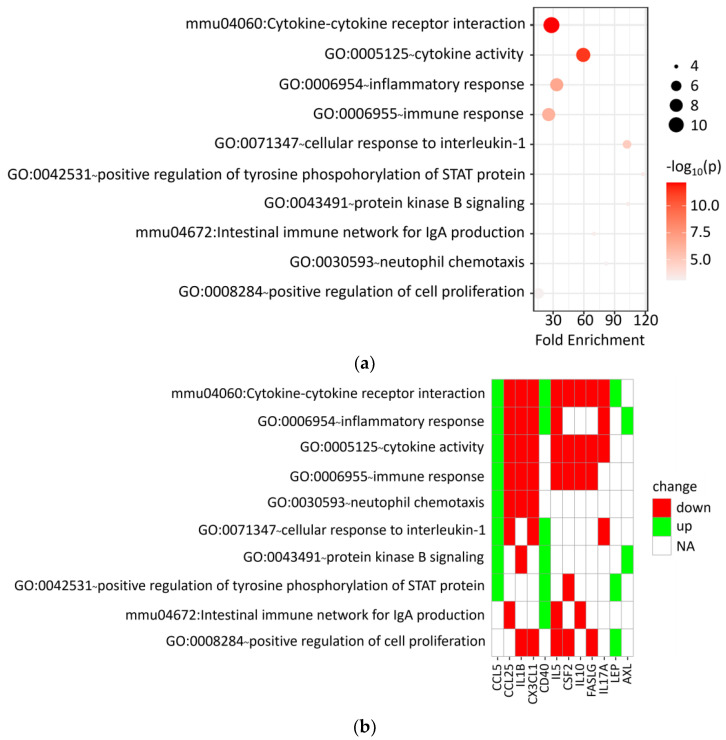
Functional enrichment analysis. Bubble chart ((**a**), upper) and heatmap ((**b**), lower) showing the top ten significant Gene Ontology terms/pathways derived from the analysis of the down-regulated (N = 9) and up-regulated (N = 5) genes in the serum samples of the two GBM groups of DVA-treated mice (oral and intraperitoneal), as compared to that of untreated controls.

Nine down-regulated (FASLG, CX3CL1, CSF2, IL1B, Il-5, IL-10, IL-17A, MIG, and CCL25) and five up-regulated (AXL, CD153, CD40, LEP, and CCL5) proteins were identified in sera from mice treated with DVA (Figure S1 Supplementary Material). No significant differences were revealed by mice treated orally or intraperitoneally. Afterwards, Gene Ontology (GO) and pathway-based analyses of genes coding for such proteins were performed using the Database for Annotation, Visualization and Integrated Discovery (DAVID), in order to provide a landscape of the induced biological response. The functional enrichment analysis of genes we considered revealed different expression profile, mainly related to cytokine–cytokine receptor interaction and modulation of the inflammatory/immune response (Figure 7). The complete list of functional annotation results is reported in Supplementary Materials Table S1.

## 4. Discussion

Our results show that the extract of DVA has a direct anticancer effect that is general in all cell lines tested: two GBM, four CRC, and three BRCA, as well as the leukaemia ones, showing in all of them a remarkable reduction in viability and a reduction in mobility in the subsample of lines tested (GBM, CRC, and BRCA are shown on Figure 2). The most impacting result (i.e., the most important for future steps towards applicability) is that both the modulation of the direct anticancer effects in live mice models of GBM and the indirect effects potentially exerted via immune system showed a two-thirds reduction in the weight of the tumour, as well as the histological evidence of damage in the remaining part. Furthermore, the gene expression showed that this effect was achieved by DVA modulating the expression profile affecting genes mainly involved in cytokine activity and inflammatory/immune response. Thus, our results, together with the small amount of published evidence both in cell cultures and in vivo models of other types of tumours, confirm the hypothesis of Landete-Castillejos et al. [18] and Wang and Landete-Castillejos [7], who have suggested that DVA may have a general anticancer effect, whereas evidence found through different assays in the experiment in mice shows how such an effect is achieved. We discuss direct effects assessed in vitro and the experiment on glioblastoma in vivo separately.

One of the strongest pieces of evidence supporting a general anticancer effect of DVA is the direct effects shown on reduction of viability and mobility in cell cultures of several types of tumours. Obviously, the evidence is widest here because, to assess direct effects on cell cultures is easier than assessing direct and indirect effects on the most complex animal models (which, on the other hand, are closer to a medical application). Our study showed further evidence of the reduction in viability of glioblastoma cancer cells on both lines (highly proliferative U87; and slower one, U251) in a dose-dependent way. This is similar to results by Chonco et al. [13] in GBM in which DVA produced a reduction of 37.5% in viability at the highest dose, whereas it did not produce such effect in non-tumour cells (HaCaT), in contrast to the damage produced by chemotherapy (temozolomide) [13]. Our results show a 36.6% (U87) and 31% (U251) reduction in viability precisely at the same dose of 1 mg/mL tested in Chonco et al.’s [13] paper. Our results show a similar reduction in viability at this dose when the DVA extract came from the middle sections of the antler (35.6% and 37.8%, respectively, for U87 and U251). Due to the close proximity of the TIP-MID sections (2.5 cm), the anti-tumour activity in other TMZ-sensitive GBM cell lines, such as U87 and U251, is an added value to our findings.

Our results provide the widest evidence published so far in a single article for the hypothesis that DVA has a general anticancer activity. DVA reduced the viability of tumour lines by 21% at 0.5 mg/mL and 25–29% at 1 mg/mL (reaching up to 49% in some cases) that were tested in colorectal and breast cancer lines DLD1, SW620, HT29, SW480, MCF7, SKBR3, and PA00. The DLD-1 cell line is particularly susceptible to the effect of the DVA compound, and this sensitivity is attributed to various molecular characteristics that influence its cellular behaviour. Among these characteristics, microsatellite instability stands out, a condition that compromises the efficiency of DNA repair. This vulnerability to the accumulation of genetic damage may enhance the response of DLD-1 to DVA treatment, as cells become less able to correct lesions in their genetic material. Furthermore, the mutation in the APC gene present also plays a crucial role in its response to DVA. The mutation in APC results in decreased regulation of key processes, such as cell division and migration. This lack of control may make DLD-1 cells more prone to DVA action, as normal cellular regulatory mechanisms are compromised. It is important to note that the presence of KRAS wild type may also contribute to its susceptibility to DVA. While activating mutations in KRAS often confer resistance to certain treatments, the wild-type version of this gene could be associated with increased receptivity to DVA [38].

The SW480 cell line shares molecular similarities with the DLD-1 cell line, including the presence of a mutation in the APC gene, microsatellite instability, and KRAS wild-type status. These characteristics are fundamental in the context of colorectal cancer, influencing the regulation of cell proliferation and genomic stability. Unlike the DLD-1 cell line, both SW480 and its metastatic derivative, SW620, exhibit notable disparities in the expression of the CD26 enzyme. This enzyme, with a significantly reduced presence in SW480 and SW620 cells, plays a crucial role in modulating substrates relevant to DVA treatment. The decrease in CD26 could influence the bioavailability of effects with anticancer action, highlighting the complexity of cellular responses to specific treatments [39].

The decreased sensitivity observed in the HT-29 cell line could be attributed in part to the presence of a mutation in the p53 gene [40]. This mutation plays a crucial role in modulating the apoptotic response to DNA damage, which could confer resistance to the induction of apoptosis and limit the efficacy of treatments that depend on this pathway. Despite sharing the feature of microsatellite instability with DLD-1 and other colorectal cell lines, instability in HT-29 is not manifested as pronounced. This variation in the degree of microsatellite instability between cell lines suggests that, although they share similarities in DNA repair capacity, the magnitude of this phenomenon may be a determining factor in the response to treatment.

In the context of breast cancer tumour lines, it is crucial to consider the distinctive molecular characteristics of MCF7, PA00, and SKBR3. MCF7, classified as luminal A, that stand out for its expression of hormone receptors, making it especially receptive to treatments that take advantage of these pathways, such as DVA, which benefits from its complex with growth hormones [41]. On the other hand, the PA00 line, classified as luminal B, presents an intriguing paradox. Despite the presence of hormone receptors, it exhibits greater proliferation and aggressiveness compared to MCF7. This phenomenon suggests a complexity in the regulation of intracellular signalling pathways, influencing their response to therapies such as DVA [42]. On the other hand, SKBR3 is characterised by the overexpression of HER2. Its absence of hormone receptors may confer resistance to DVA compared to luminal lines. However, overexpression of HER2 gives it an exceptional ability to invade tissues [43]. More interestingly, it was effective in both primary tumours and secondary ones, in those sensitive to chemotherapy, and in those multi-resistant to it. In contrast, and in line with evidence of our previous study in GBM [13], DVA had no significant general reduction in viability in non-cancerous lines 293T and HaCaT, except in one case for each line, only from one of the two sections at the highest dose. Considering this has not been found either in non-tumour cells in the study of Chonco et al. [13] or Yang et al. [11], it is reasonable to conclude that DVA does not reduce viability in healthy cells. In addition to the evidence previously published in GBM lines T98 and A172, the study by Yang et al. [11] showed that DVA (here tested as sika deer growing antler protein) is as effective as chemotherapy in cell cultures of prostate cancer. This is very interesting, as the growing antler has tissues such as skin, cartilage, bone, blood vessels, and nerves growing, but not like those in a prostate.

DVA also reduced the mobility of both U87 and U251 GBM cell lines. This effect is cumulative (i.e., stronger at 48 than at 24 h) so that cells did not compensate after an initial slow down (it does not disappear on the second day tested). Not only that, but the effect was greater at 48 h (greater difference with control) for DVA-TIP. In the study of Chonco et al. [13], who also examined DVA-TIP at 1 mg/mL in a scratch test, T98 GBM lines reduced their mobility by 39% at 6 h, whereas it had no effect on healthy cells (HaCaT). As with viability, the results found here in other types of tumours (colorectal and breast cancer lines) for DVA also showed a reduced mobility that was clearer in some lines over others, but the most general effect was the tip reduction in mobility at 72 h.

Considering that, as shown by Cao et al. [17] and Li et al. [15] in other types of tumours, the effects of DVA or its derived proteins exert part of their effects via immune system cells and organs (such as spleen and thymus) [15,17], we tested whether DVA could reduce the viability of tumour cells precisely in those originated from immune cells: THP-1 monocytes and differentiated macrophages (M0). Our prediction was confirmed by results with similar 40% reduction in viability in both cases (a figure very similar to that of Chonco et al. [13], in GBM). This result has also potential implications to explain the effect found in tumours in mice: as indicated in the introduction, M0 can be polarised into TAMs, of which M2 is responsible for a great part of the immune system tolerance to the tumour. DVA may reduce the M0 that have infiltrated into the tumour, killing a proportion of them and potentially reducing the M2 available to protect the tumour from M1 and cytotoxic T cells.

The assays on the M1/M2 environment showed a reduction in GBM viability with both types of macrophages. Actually, the reduction in viability was greater than if DVA was added, although DVA also had, as in previous tests, an effect. The surprising fact is that M2 induces a tolerant effect towards the tumour. Genin et al. (2015) found that co-cultures of THP-1-M2 macrophages and HepG2 or A549 cancer cells reduced the level of chemotherapy-induced apoptosis in these cancer cell lines, whereas THP-1-M1 macrophages increased the cell death above the chemotherapy alone [29]. In our case, DVA showed an unexpected protective effect in co-cultures with M2 with respect to the effect of M2 alone (despite the fact that DVA alone kills tumour cells), whereas for M1, there is an apparent synergistic effect of DVA and M1 to reduce further the viability, but at the small sample size used, this was not significant. However, in the complex environment of the tumour, with cytotoxic T cells infiltrating the tumour to kill it and a whole array of cytokines and immune cells interacting, the size of the effect and synergies of DVA may be different (as the results in vivo further below suggest).

All the above-mentioned evidence on GBM cell lines adds to published studies on other direct effects produced by DVA: thus, Chonco et al. [13] found an 84% reduction in colony formation capacity, much greater than the 40% achieved with temozolomide at the lower dose 20 μg/mL and somewhat similar to the 95% achieved at the high dose of 200 μg/mL. This paper suggests that DVA could promote apoptosis in GBM, as the authors found a non-significant trend of a 3-fold increase in the number of apoptotic cells. The effect was confirmed in other types of tumours as Cao et al. [17] found, in mice with sarcoma, that apoptotic tumour cells increased from 6% (untreated mice with sarcoma) to 76% (at the highest DVA dose), whereas the number of normal sarcoma cells decreased from 91% (untreated) to 19% (highest DVA dose) [17]. Obviously, in a living mouse, the apoptosis may be produced by several mechanisms (including the apoptosis induced by cytotoxic T cells), whereas in a cell plate, the DVA effect can only be direct, but results in both studies suggest that DVA induce apoptosis in the tumours tested. Why would DVA induce high levels of apoptosis? In a very recent paper, Li et al. [15] proposed that the possible strategy to prevent tumour growth in antlers is the highly efficient cell apoptosis mechanism, and that this would be the reason why inner (IR) layer of the reserve mesenchyme in the antler tip (the layer proliferating fastest in the antler) shows a 64% level of apoptosis, which is the highest in the antler and higher than in any other adult tissue [20]. Thus, our hypothesis here is that at least one of the direct anticancer effects of DVA on tumour cells is the use of the highly effective signalling of apoptosis (evolved for the development of the antler to induce apoptosis specifically in the tumour cells). This points to a very interesting line of research to fight cancer, as the induced cell death is specifically aimed at cancer cells and not healthy proliferating cells.

Some of the most interesting results were those produced in the experiment with the xenograft model. Previous research had shown similar effects of a dose of 200 and 400 mg/kg live mice, inhibiting around 65% the growth in weight or volume of the human prostate cancer xenograft [12]. That is why we selected this dose in our experiment. We tried two administrations, expecting that the oral one would show either very reduced or no effects of DVA on GBM tumour. The results show a reduction in tumour weight surprisingly similar to that in the study of Tang et al. [12]: 66% reduction, despite using another cervid species (sika deer, *Cervus nippon* vs. *Cervus elaphus* here). To ensure comparability, we prepared the samples freeze-dried and milled in a similar way to Tang et al. [12]. Contrary to our expectations, the oral administration produced an effect rather similar to the intraperitoneal one (only a slight reduction of 4.9 percent units less than intraperitoneal administration), despite the fact that DVA was digested in the former. Interestingly, the study by Cao et al. [17], which used only one protein from the remains of producing a velvet antler alcoholic drink (called antler wine in China), the VA-pro (acronym for velvet antler protein) showed a reduction of mice sarcoma not very far from the data shown above: 55% reduction at the 100 mg/kg dose. And this, despite the treatment, was given for a shorter time (16 vs. 28 days in our study) and, more importantly, as a preventive treatment before the sarcoma was introduced [17]. A similar 50% reduction was produced by DVA in Li et al. [15] in mice growing another type of human tumour: triple-negative breast cancer. This occurred despite using a commercial powder of DVA, which likely included the whole antler (as top sections reach a much higher price in the market [44]); therefore, it should be less effective. Thus, DVA seems to have a wide and strong anticancer effect in live mice too, whether they have their full immune system, as in the work of authors such as in Cao et al. [17]), or as in our case, Tang et al.’s case [12], and Li et al.’s case [15], partly compromised in order to grow a human tumour.

Despite using the nude mice to be able to grow a human tumour, the results show evidence of DVA effects in the immune system to the partial extent that using this strain of mice can show. Nude mice do not have thymus, and therefore, our experiment cannot show the effects of DVA on this organ as it showed in the study by Cao et al. [17]. However, they do have a spleen, and the results showed a 44% and 30% reduction in spleen size when DVA was administered IP or orally, respectively. As indicated in the introduction, spleen weight varies with tumour progression and may be a predictor of tumour recurrence. In tumour-bearing mice (which is our case), the spleen generates immuno-suppressive myeloid cells [31,32] that are also involved in inducing immune tolerance towards the tumour [21,33]. Spleen is also involved in the proliferation of monocytes that infiltrate the tumour [27] and then differentiate into macrophages (called M0), that may polarise into M1 or M2. Either if spleen grows to produce more cells that attack the tumour, or it is induced by the tumour to produce more cells to protect it, the result is that, as we found in our study, the greatest variability explained by spleen size is tumour size. Thus, the reduction in tumour produced by DVA results in smaller spleen size. Cao et al. [17] also showed that the greater the dose of DVA, the smaller the spleen and the closer its weight to that of the healthy mice (whereas the greatest spleen was that of control mice with tumour).

The effect of DVA on the tumour was further clarified by the histochemistry and the assays on differential gene expression profiles. The microscopy exam showed evidence of direct effects of DVA inducing necrosis directly on tumour cells, as well as in the supportive tissue, such as in blood vessels. Part of such direct effect may be achieved by the activity of immune cells. Although, due to the lack of a thymus, nude mice cannot generate mature T lymphocytes and therefore are unable to carry out many types of adaptive immune responses, it must be considered that most of the nude mouse strains used are slightly immunosuppressed and have some T cells, especially as they age. The histopathological results of the study by Li et al. [15] examining the effect of DVA on human triple-negative breast cancer treated with chemotherapy (neoadjuvant or NAC) also showed, as in our study, signs of cell death at the tissue level [15]. This DVA effect appears to have been achieved by promoting the immune system because the CD4^+^ and CD8^+^ T cell numbers, when DVA was added to NAC treatment in the study by Li et al., were higher than numbers in mice with tumours or those treated with NAC without DVA. Unfortunately, we did not measure numbers of CD4^+^ or CD8^+^ T cells in our study to confirm an increase in numbers when treated with DVA.

In any case, we believe that part of the anti-tumour effect of DVA could have been exerted through changes in the tissue microenvironment in which immune cells can localise. We know that increasing numbers of tumour-infiltrating TAMs is correlated to poor survival among recurrent GBM patients [45]. Thus, DVA would reduce the tumour as a mixture of different actions: killing the cancer cells, reducing the tumour-associated macrophages M2 (or also repolarizing them into M1 killers), reducing myeloid-derived suppressor cells (MDSCs) and tumour-associated neutrophils (TANs) that grow to create this permissive immune environment for the tumour [24,25,26], and increasing the numbers of T killer cells found by Cao et al. [17] and Li et al. [15]. Certainly, the reduction of viability of both monocytes and related macrophages from tumour cell lines produced by DVA in this study may explain the effects on the GBM xenograft mouse model found.

Also, we analysed the concentration of cytokines from the serum of the mice to understand how DVA may influence the inflammatory state of the tumour. The results showed a down-regulation of some factors after the treatment with DVA extract, like FasL, which is associated with the increase of tumour progression [46]; id est, DVA produces the opposite effect. Other chemokines found down-regulated and for which, therefore, DVA reduces their functions are CX3CL1 (Fractalkine), which with its axis stimulates cancer cell migration [47]; GM-CSF, which, when it is very high, can exhaust immune cells and promote cancer growth [48]; and some ILs, such as IL1-b, which is able to induce angiogenesis and cancer cell proliferation [49]. We also found that DVA produced up-regulation of some chemokines which constitute promising targets for cancer immunotherapy. One of them, for example, was CD30L, which is a molecule that regulated the glioma microenvironment so that, when it is deficient (i.e., the opposite effect produced by DVA), it leads to a pro-tumorigenic phenotype [50]. Another interesting result is that DVA enhanced the expression of CD40, which seems to produce anti-tumour effects in several tumour models [51]. Altogether, these findings show that DVA induces a different expression profile in GBM mouse models, producing an interference with cytokine–cytokine receptor interaction and activity that would be part of the normal progression of GBM tumour had the mice not been treated with DVA. As a consequence, this causes a different immune response in treated mice that modulates the inflammatory response regulating the tumoral micro-environment in a way that leads to anti-proliferative tumour conditions.

### The Potential of DVA or Its Molecules as a Future Medicine against Cancer

DVA does not have the properties required to become a modern medicine, as it is a complex mixture of molecules, some of which are active against cancer, whereas minerals and other molecules may not have effects on health. Furthermore, as a biological extract, it is variable in composition depending on some factors we know affect it (stage of growth, sections of the antler used to create the extract, age of the deer, and probably even size of the antler) and others that we do not know (there appears to be inter-individual differences in the efficiency of the anticancer effect). A medicine should have one or few active principles of fully known effects (both positive on health and its secondary effects), and its content per pill or dose should be clearly defined and without variability. Thus, the final aim of this research line should be to find such a molecule or small set of molecules within DVA that have anticancer properties to develop a future medicine that may have wide anticancer activity without secondary effects. What are the candidates for such molecule? According to Sunwoo et al. [52], the tip of the growing antler is composed mostly of protein (69%), followed by lipids (19%), and most of the rest is ash (minerals). The most likely candidate for an active molecule, based on the fact that is the most abundant, as well as soluble in water (the solvent most often used), is a protein. However, other types of molecules cannot be discarded, as a minor component may be very powerful against cancer despite being in small quantities. Studies assessing anticancer properties have focused on proteins. Thus, Cao et al. [17] isolated from the velvet antler by-product of antler wine a 23.088 kDa protein that had the anticancer and immune system-promoting effects reported in this study. In a rather coarser purification method, Yang et al. [11] extracted, with 50% water/ethanol solvent, from DVA powder a set of proteins ranging from 250 kDa to 35 kDa that they called Sika Deer Antler Protein. The 23 kDa protein of Cao et al. [17] appears to be outside this range, but it should be noted that, in the first case, the species is the red deer, and in the second, it is the sika deer. Equally, the study by Li et al. [15] was a set of soluble proteins of unknown size; however, in this case, they were dissolved in water.

## 5. Conclusions

The extract from the growing antler (DVA) showed wide and direct anticancer effects in several lines of cell cultures, both of primary tumours sensitive to chemotherapy and those from relapsing ones and multi-resistant to therapy belonging to four types of tumours: glioblastoma, colorectal, breast cancer, and leukaemia. The scratch tests showed that DVA, in general, also reduced the mobility of the cancer cell lines tested. The most applicable results were the effects of DVA in reducing glioblastoma tumour in nude mice, where it showed a 61–66% reduction in tumour weight, which was parallel to the reduction in weight of the spleen (43.8%). The histopathological exam showed that the remaining DVA-treated tumour showed necrosis both in the cancer cells and in the supporting vessels. Altogether, the reduction in tumour and spleen and the damage revealed at microscopic level, as the up-regulation of anticancer genes, as well as the down-regulation of those promoting cancer tolerance, show that DVA had both direct effects and indirect effects mediated by the immune system. This is the first single study showing comprehensive evidence using the same methods on several cancer types and several cell lines per type of cancer, and on general anticancer effects of the extract of growing antler, as well as offering insight into the mechanisms by which this is achieved in animal models (with human glioblastoma as an example). Although many questions remain unanswered, the anticancer effects of DVA found here may lead to the finding of one or more of its molecules that may become a future widely effective medicine without secondary effects. It seems likely that part of this efficiency will be achieved by promoting the immune system to fight cancer or reducing the tolerance induced by the tumour.

## Data Availability

The scientific staff of the universities involved in this project have plans of data management. UCLM: https://www.uclm.es/Areas/Biblioteca/Investiga/OpenScience/OpenData. CTB: https://drive.upm.es/s/y6P4yCp4GXKfmfv (this one contains the data of the current paper; accessed on 30 April 2024). The data does not contain information of human patients, so there was no specific requirement for this type of data. All data are available in the main text or the Supplementary Materials.

## Supplementary Materials

The following supporting information can be downloaded at https://www.mdpi.com/article/10.3390/pharmaceutics16050610/s1, Table S1: List of functional annotation; Figure S1: Mouse cytokine antibody array; Figure S2: Images of scratch assay; Figure S3. Images of xenograft tumours.

## References

[1] Sung H., Ferlay J., Siegel R.L., Laversanne M., Soerjomataram I., Jemal A., Bray F. (2021). Global Cancer Statistics 2020: GLOBOCAN Estimates of Incidence and Mortality Worldwide for 36 Cancers in 185 Countries. CA Cancer J. Clin..

[2] Gaspar-López E., Landete-Castillejos T., Estevez J.A., Ceacero F., Gallego L., García A.J. (2010). Biometrics, Testosterone, Cortisol and Antler Growth Cycle in Iberian Red Deer Stags (*Cervus elaphus hispanicus*). Reprod. Domest. Anim..

[3] Gomez S., Garcia A.J., Luna S., Kierdorf U., Kierdorf H., Gallego L., Landete-Castillejos T. (2013). Labeling Studies on Cortical Bone Formation in the Antlers of Red Deer (*Cervus elaphus*). Bone.

[4] Landete-Castillejos T., Kierdorf H., Gomez S., Luna S., García A.J., Cappelli J., Pérez-Serrano M., Pérez-Barbería J., Gallego L., Kierdorf U. (2019). Antlers-Evolution, Development, Structure, Composition, and Biomechanics of an Outstanding Type of Bone. Bone.

[5] Valerius G. (1998). Deer of the World, Their Evolution, Behaviour, and Ecology.

[6] Goss R.J. (1970). Problems of Antlerogenesis. Clin. Orthop. Rel. Res..

[7] Wang D., Landete-Castillejos T. (2023). Stem Cells Drive Antler Regeneration. Science.

[8] Wang Y., Zhang C., Wang N., Li Z., Heller R., Liu R., Zhao Y., Han J., Pan X., Zheng Z. (2019). Genetic Basis of Ruminant Headgear and Rapid Antler Regeneration. Science.

[9] Fan Y.L., Xing Z., Wei Q., Liu G.R., Li H.P., Zhou S.Q. (1998). A Study on the Extraction Separation and Anticancer Activity of Velvet Antler Protein. J. Econ. Anim..

[10] Xiong H.L. (2007). Extraction and Isolation of Activity Component from Velvet Antler and Research of Its Anti-Tumor Effect. Master’s Thesis.

[11] Yang H., Wang L., Sun H., He X., Zhang J., Liu F. (2017). Anticancer Activity in Vitro and Biological Safety Evaluation in Vivo of Sika Deer Antler Protein. J. Food Biochem..

[12] Tang Y., Fan M., Choi Y.-J., Yu Y., Yao G., Deng Y., Moon S.-H., Kim E.-K. (2019). Sika Deer (*Cervus nippon*) Velvet Antler Extract Attenuates Prostate Cancer in Xenograft Model. Biosci. Biotechnol. Biochem..

[13] Chonco L., Landete-Castillejos T., Serrano-Heras G., Serrano M.P., Pérez-Barbería F.J., González-Armesto C., García A., de Cabo C., Lorenzo J.M., Li C. (2021). Anti-Tumour Activity of Deer Growing Antlers and Its Potential Applications in the Treatment of Malignant Gliomas. Sci. Rep..

[14] Hu W., Qi L., Tian Y.H., Hu R., Wu L., Meng X.Y. (2015). Studies on the Purification of Polypeptide from Sika Antler Plate and Activities of Antitumor. BMC Complement. Altern. Med..

[15] Li M., Li Q., Dong H., Zhao S., Ning J., Bai X., Yue X., Xie A. (2022). Pilose Antler Polypeptides Enhance Chemotherapy Effects in Triple-Negative Breast Cancer by Activating the Adaptive Immune System. Int. J. Biol. Macromol..

[16] Fraser A., Haines S.R., Stuart E.C., Scandlyn M.J., Alexander A., Somers-Edgar T.J., Rosengren R.J. (2010). Deer Velvet Supplementation Decreases the Grade and Metastasis of Azoxymethane-Induced Colon Cancer in the Male Rat. Food Chem. Toxicol..

[17] Cao T.-Q., An H.-X., Ma R.-J., Dai K.-Y., Ji H.-Y., Liu A.-J., Zhou J.-P. (2023). Structural Characteristics of a Low Molecular Weight Velvet Antler Protein and the Anti-Tumor Activity on S180 Tumor-Bearing Mice. Bioorg. Chem..

[18] Landete-Castillejos T., Rossetti A., Garcia A.J., de Cabo C., Festuccia C., Luna S., Chonco L. (2022). From a General Anti-Cancer Treatment to Antioxidant or Deer Osteoporosis: The Consequences of Antler as the Fastest-Growing Tissue. Anim. Prod. Sci..

[19] Liu L., Jiao Y., Yang M., Wu L., Long G., Hu W. (2023). Network Pharmacology, Molecular Docking and Molecular Dynamics to Explore the Potential Immunomodulatory Mechanisms of Deer Antler. Int. J. Mol. Sci..

[20] Li C., Li Y., Wang W., Scimeca M., Melino G., Du R., Shi Y. (2023). Deer Antlers: The Fastest Growing Tissue with Least Cancer Occurrence. Cell Death Differ..

[21] Ugel S., Peranzoni E., Desantis G., Chioda M., Walter S., Weinschenk T., Ochando J.C., Cabrelle A., Mandruzzato S., Bronte V. (2012). Immune Tolerance to Tumor Antigens Occurs in a Specialized Environment of the Spleen. Cell Rep..

[22] Yang M., Pu L., Yang S., Chen Z., Guo J., Liu Y., Chang S., Peng Y. (2024). Engineered Antler Stem Cells Derived Exosomes Potentiate Anti-Tumor Efficacy of Immune Checkpoint Inhibitor by Reprogramming Immunosuppressive Tumor Microenvironment. Chem. Eng. J..

[23] Zitvogel L., Apetoh L., Ghiringhelli F., Kroemer G. (2008). Immunological Aspects of Cancer Chemotherapy. Nat. Rev. Immunol..

[24] Marvel D., Gabrilovich D.I. (2015). Myeloid-Derived Suppressor Cells in the Tumor Microenvironment: Expect the Unexpected. J. Clin. Investig..

[25] Xia Y., Wei Y., Li Z.-Y., Cai X.-Y., Zhang L.-L., Dong X.-R., Zhang S., Zhang R.-G., Meng R., Zhu F. (2019). Catecholamines Contribute to the Neovascularization of Lung Cancer via Tumor-Associated Macrophages. Brain Behav. Immun..

[26] Jiang W., Li Y., Wei W., Li J.-W., Li L., Zhang C., Zhang S.-Q., Kong G.-Y., Li Z.-F. (2020). Spleen Contributes to Restraint Stress Induced Hepatocellular Carcinoma Progression. Int. Immunopharmacol..

[27] Pollard J.W. (2004). Tumour-Educated Macrophages Promote Tumour Progression and Metastasis. Nat. Rev. Cancer.

[28] Liu L., Wang Y., Guo X., Zhao J., Zhou S. (2020). A Biomimetic Polymer Magnetic Nanocarrier Polarizing Tumor-Associated Macrophages for Potentiating Immunotherapy. Small.

[29] Genin M., Clement F., Fattaccioli A., Raes M., Michiels C. (2015). M1 and M2 Macrophages Derived from THP-1 Cells Differentially Modulate the Response of Cancer Cells to Etoposide. BMC Cancer.

[30] Han S., Bao X., Zou Y., Wang L., Li Y., Yang L., Liao A., Zhang X., Jiang X., Liang D. (2023). D-Lactate Modulates M2 Tumor-Associated Macrophages and Remodels Immunosuppressive Tumor Microenvironment for Hepatocellular Carcinoma. Sci. Adv..

[31] Cortez-Retamozo V., Etzrodt M., Newton A., Rauch P.J., Chudnovskiy A., Berger C., Ryan R.J.H., Iwamoto Y., Marinelli B., Gorbatov R. (2012). Origins of Tumor-Associated Macrophages and Neutrophils. Proc. Natl. Acad. Sci. USA.

[32] Cortez-Retamozo V., Etzrodt M., Newton A., Ryan R., Pucci F., Sio S.W., Kuswanto W., Rauch P.J., Chudnovskiy A., Iwamoto Y. (2013). Angiotensin II Drives the Production of Tumor-Promoting Macrophages. Immunity.

[33] Wu C., Ning H., Liu M., Lin J., Luo S., Zhu W., Xu J., Wu W.-C., Liang J., Shao C.-K. (2018). Spleen Mediates a Distinct Hematopoietic Progenitor Response Supporting Tumor-Promoting Myelopoiesis. J. Clin. Investig..

[34] Kumar V., Patel S., Tcyganov E., Gabrilovich D.I. (2016). The Nature of Myeloid-Derived Suppressor Cells in the Tumor Microenvironment. Trends Immunol..

[35] Muir P.D., Sykes A.R., Barrell G.K. (1987). Growth and mineralisation of antlers in red deer (*Cervus elaphus*). N. Z. J. Agric. Res..

[36] García Bueno J.M., Ocaña A., Castro-García P., Gil Gas C., Sánchez-Sánchez F., Poblet E., Serrano R., Calero R., Ramírez-Castillejo C. (2008). An Update on the Biology of Cancer Stem Cells in Breast Cancer. Clin. Transl. Oncol..

[37] Gundara J.S., Gill A.J., Samra J.S. (2015). Efficacy of Primary Tumour Volume as a Predictor of Survival Compared with Size Alone in Pancreatic Ductal Adenocarcinoma. Oncol. Lett..

[38] Sánchez-Díez M., Alegría-Aravena N., López-Montes M., Quiroz-Troncoso J., González-Martos R., Menéndez-Rey A., Sánchez-Sánchez J.L., Pastor J.M., Ramírez-Castillejo C. (2022). Implication of Different Tumor Biomarkers in Drug Resistance and Invasiveness in Primary and Metastatic Colorectal Cancer Cell Lines. Biomedicines.

[39] Cordero O.J. (2022). CD26 and Cancer. Cancers.

[40] Chee C.W., Mohd Hashim N., Nor Rashid N. (2024). Morindone as a Potential Therapeutic Compound Targeting TP53 and KRAS Mutations in Colorectal Cancer Cells. Chem. Biol. Interact..

[41] Marks M.P., Giménez C.A., Isaja L., Vera M.B., Borzone F.R., Pereyra-Bonnet F., Romorini L., Videla-Richardson G.A., Chasseing N.A., Calvo J.C. (2024). Role of Hydroxymethylglutharyl-Coenzyme A Reductase in the Induction of Stem-like States in Breast Cancer. J. Cancer Res. Clin. Oncol..

[42] Gil-Gas C., Sánchez-Díez M., Honrubia-Gómez P., Sánchez-Sánchez J.L., Alvarez-Simón C.B., Sabater S., Sánchez-Sánchez F., Ramírez-Castillejo C. (2023). Self-Renewal Inhibition in Breast Cancer Stem Cells: Moonlight Role of PEDF in Breast Cancer. Cancers.

[43] Zuben de Valega Negrão C.V., Cerize N.N., da Silva Justo-Junior A., Liszbinski R.B., Meneguetti G.P., Araujo L., Rocco S.A., de Almeida Gonçalves K., Cornejo D.R., Leo P. (2024). HER2 Aptamer-Conjugated Iron Oxide Nanoparticles with PDMAEMA-b-PMPC Coating for Breast Cancer Cell Identification. Nanomedicine.

[44] Serrano M.P., Maggiolino A., Pateiro M., Landete-Castillejos T., Domínguez R., García A., Franco D., Gallego L., De Palo P., Lorenzo J.M., Lorenzo J.M., Munekata P.E.S., Barba F.J., Toldrá F. (2019). Carcass Characteristics and Meat Quality of Deer. More Than Beef, Pork and Chicken—The Production, Processing, and Quality Traits of Other Sources of Meat for Human Diet.

[45] Cui X., Morales R.-T.T., Qian W., Wang H., Gagner J.-P., Dolgalev I., Placantonakis D., Zagzag D., Cimmino L., Snuderl M. (2018). Hacking Macrophage-Associated Immunosuppression for Regulating Glioblastoma Angiogenesis. Biomaterials.

[46] Kim R., Emi M., Tanabe K., Uchida Y., Toge T. (2004). The Role of Fas Ligand and Transforming Growth Factor β in Tumor Progression. Cancer.

[47] Korbecki J., Simińska D., Kojder K., Grochans S., Gutowska I., Chlubek D., Baranowska-Bosiacka I. (2020). Fractalkine/CX3CL1 in Neoplastic Processes. Int. J. Mol. Sci..

[48] Kumar A., Taghi Khani A., Sanchez Ortiz A., Swaminathan S. (2022). GM-CSF: A Double-Edged Sword in Cancer Immunotherapy. Front. Immunol..

[49] Rébé C., Ghiringhelli F. (2020). Interleukin-1β and Cancer. Cancers.

[50] Duan J., Gao Y., Zhang X., Wang X., Wang B., Meng X., Yoshikai Y., Wang Y., Sun X. (2019). CD30 Ligand Deficiency Accelerates Glioma Progression by Promoting the Formation of Tumor Immune Microenvironment. Int. Immunopharmacol..

[51] Djureinovic D., Wang M., Kluger H.M. (2021). Agonistic CD40 Antibodies in Cancer Treatment. Cancers.

[52] Sunwoo H.H., Nakano T., Hudson R.J., Sim J.S. (1995). Chemical composition of antlers from Wapiti (*Cervus elaphus*). J. Agric. Food Chem..

