# Supplementary material for: General Direct Anticancer Effects of Deer Growing Antler Extract in Several Tumour Cell Lines, and Immune System-Mediated Effects in Xenograft Glioblastoma
Source: Pharmaceutics. 2024 Apr 30;16(5):610. doi: 10.3390/pharmaceutics16050610 (PMC11125008; doi:10.3390/pharmaceutics16050610)
Supplement: Supplementary file 1 [file pharmaceutics-16-00610-s001.zip › pharmaceutics-2953024-supplementary.pdf]

Supplementary Materials for General Direct Anticancer Effects of Deer Growing Antler Extract in Several Tumour Cell Lines, and Immune System-Mediated Effects in Xenograft Glioblastoma

Alessandra Rossetti *et al.*

Corresponding author: Tomás Landete Castillejos, Tomas.Landete@uclm.es

This PDF file includes the following:

Figure S1,
Figure S2,
Figure S3,
Table S1.

Figure S1. Mouse cytokine antibody array.

(a)

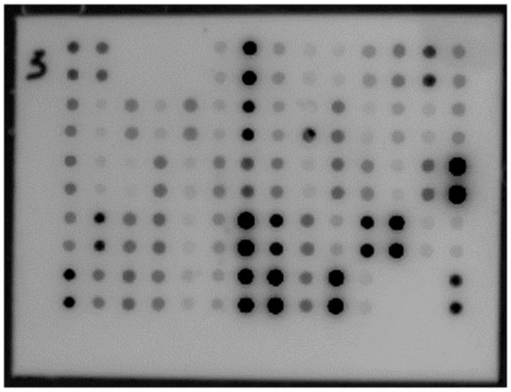

	A	B	C	D	E	F	G	H	I	J	K	L	M	N
1 2	POS	POS	NEG	NEG	BLANK	Axl	BLC (CXCL13)	CD30 Ligand (TNFSF8)	CD30 (TNFRSF8)	CD40 (TNFRSF5)	CRG-2	CTACK (CCL27)	CXCL16	Eotaxin-1 (CCL11)
3 4	Eotaxin-2 (CCL24)	Fas Ligand (TNFSF6)	Fractalkine (CX3CL1)	GCSF	GM-CSF	INF-γ	IGFBP-3	IGFBP-5	IGFBP-6	IL-1α (IL-1 F1)	IL-1β (IL-1 F2)	IL-2	IL-3	IL-3 Rβ
5 6	IL-4	IL-5	IL-6	IL-9	IL-10	IL-12 p40/p70	IL-12 p70	IL-13	IL-17A	KC (CXCL1)	Leptin R	Leptin	LIX	L-Selectin (CD62L)
7 8	Ltn (XCL1)	MCP-1 (CCL2)	MCP-5	M-CSF	MIG (CXCL9)	MIP-1α (CCL3)	MIP-1 γ	MIP-2	MIP-3β (CCL19)	MIP-3α (CCL20)	PF-4 (CXCL4)	P-Selectin	RANTES (CCL5)	SCF
9 10	SDF-1 α	TARC (CCL17)	I-309 (TCA-3/CCL1)	TECK (CCL25)	TIMP-1	TNFα	TNR RI (TNRFRSF1A)	TNF RII (TNFRSF 1B)	TPO	VCAM-1 (CD106)	VEGF-A	BLANK	BLANK	POS

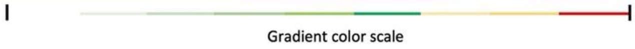

(b)

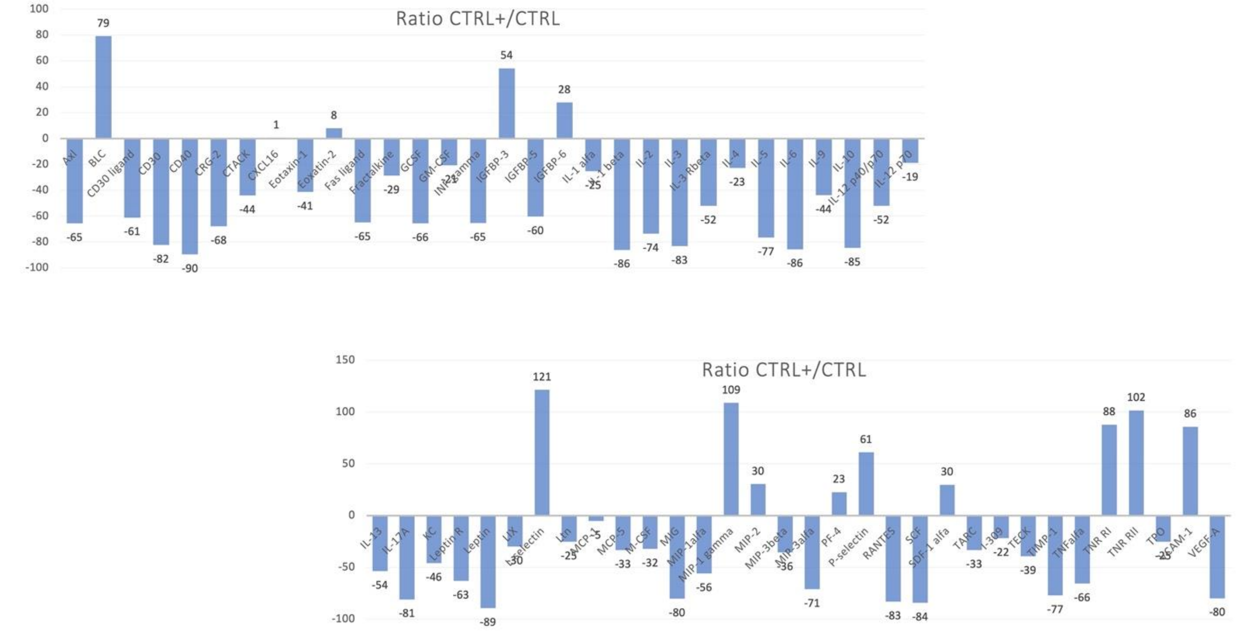

(c)

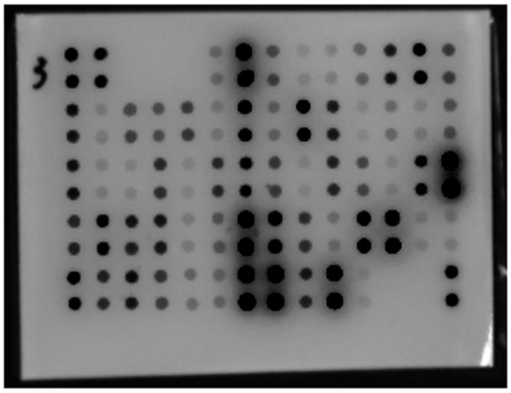

	A	B	C	D	E	F	G	H	I	J	K	L	M	N
1 2	POS	POS	NEG	NEG	BLANK	Axl	BLC (CXCL13)	CD30 Ligand (TNFSF8)	CD30 (TNFRSF8)	CD40 (TNFRSF5)	CRG-2	CTACK (CCL27)	CXCL16	Eotaxin-1 (CCL11)
3 4	Eotaxin-2 (CCL24)	Fas Ligand (TNFSF6)	Fractalkine (CX3CL1)	GCSF	GM-CSF	INF-γ	IGFBP-3	IGFBP-5	IGFBP-6	IL-1α (IL-1 F1)	IL-1β (IL-1 F2)	IL-2	IL-3	IL-3 Rβ
5 6	IL-4	IL-5	IL-6	IL-9	IL-10	IL-12 p40/p70	IL-12 p70	IL-13	IL-17A	KC (CXCL1)	Leptin R	Leptin	LIX	L-Selectin (CD62L)
7 8	Ltn (XCL1)	MCP-1 (CCL2)	MCP-5	M-CSF	MIG (CXCL9)	MIP-1α (CCL3)	MIP-1 γ	MIP-2	MIP-3β (CCL19)	MIP-3α (CCL20)	PF-4 (CXCL4)	P-Selectin	RANTES (CCL5)	SCF
9 10	SDF-1 α	TARC (CCL17)	I-309 (TCA-3/CCL1)	TECK (CCL25)	TIMP-1	TNFα	TNR RI (TNRFRSF1A)	TNF RII (TNFRSF 1B)	TPO	VCAM-1 (CD106)	VEGF-A	BLANK	BLANK	POS

Gradient color scale

(d)

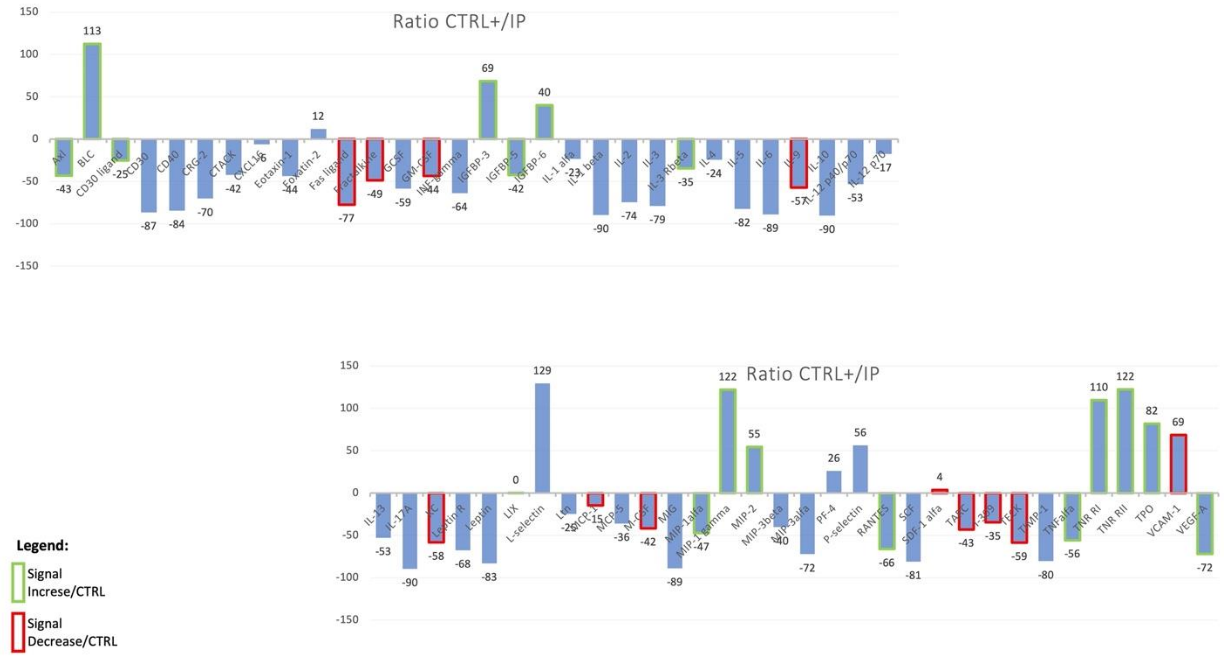

Mouse Cytokine Antibody Array C3 was used for evaluating differentially expressed cytokines in sera from DVA treated mice: (a) photo of the membrane incubated with serum of CTRL group, with table of related cytokines expression; (b) histograms about the percentage of proteins expression of CTRL sample compared to the positive control; (c) photo of the membrane incubated with serum of IP treated group, with table of related cytokines expression; and (d) histograms about the percentage of proteins expression of IP sample compared to positive control. This graph underlines that cytokines are more or less expressed than CTRL. Two technical and two biological replicates were performed.

Figure S2. Images of scratch assay.

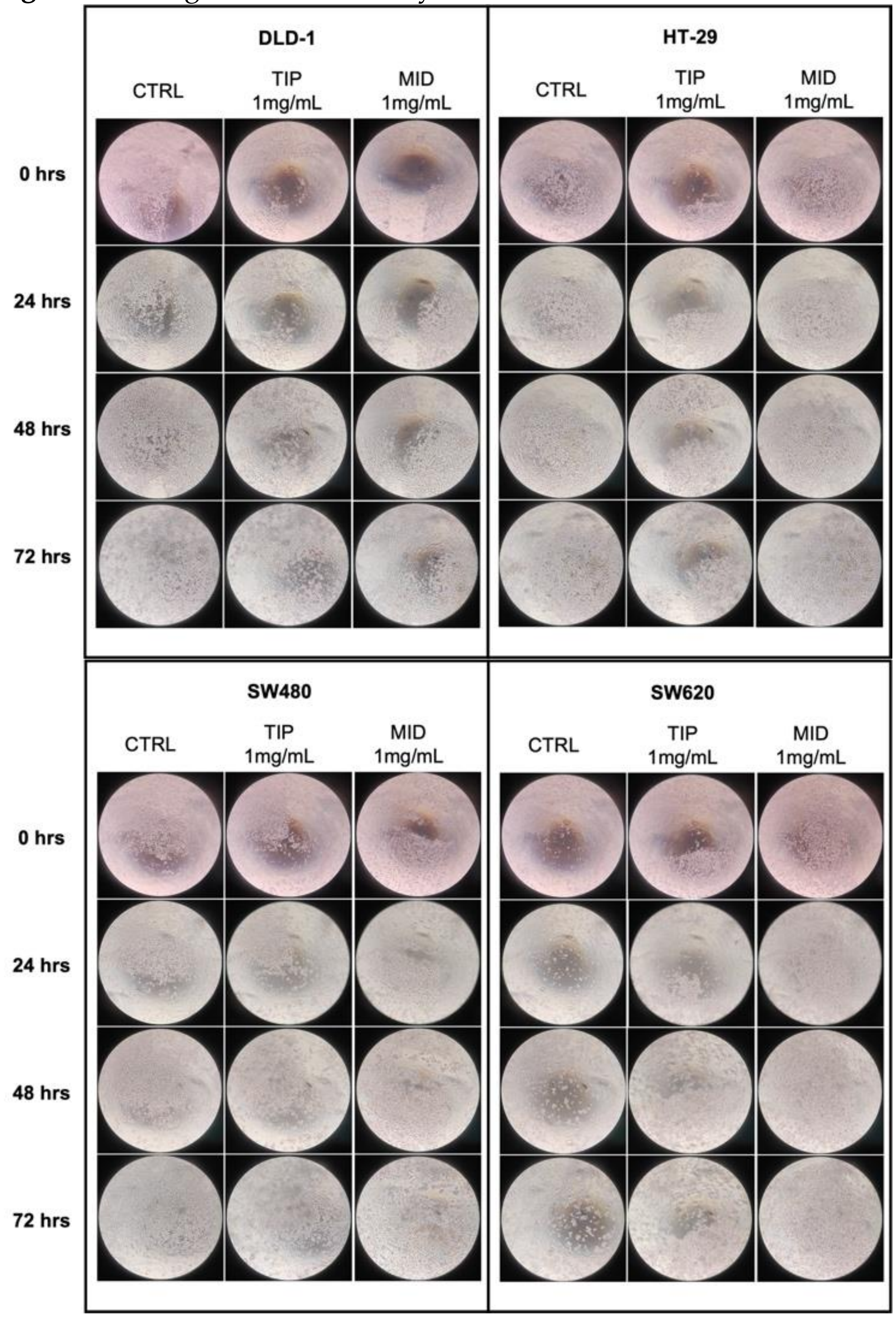

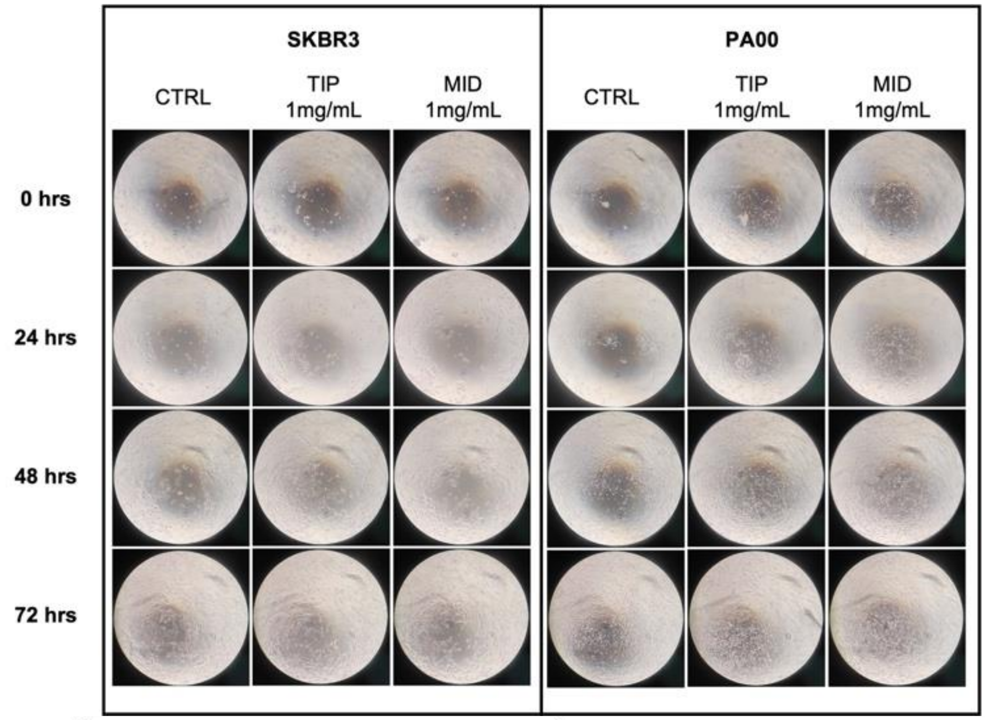
SKBR3
PA00
CTRL
TIP 1mg/mL
MID 1mg/mL
CTRL
TIP 1mg/mL
MID 1mg/mL
0 hrs
24 hrs
48 hrs
72 hrs

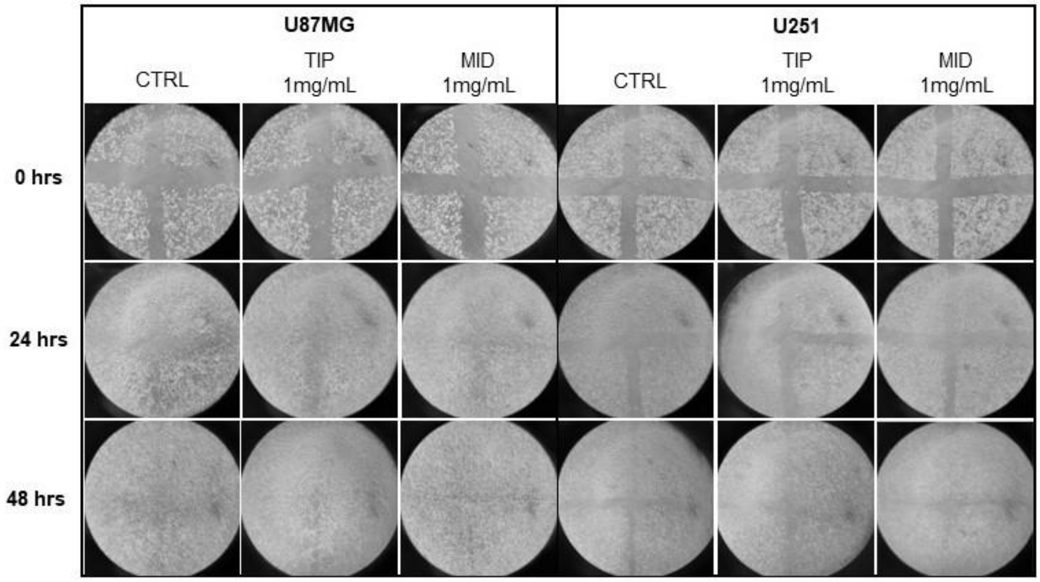
U87MG
U251
CTRL
TIP 1mg/mL
MID 1mg/mL
CTRL
TIP 1mg/mL
MID 1mg/mL
0 hrs
24 hrs
48 hrs

Figure S3. Images of xenograft tumours.

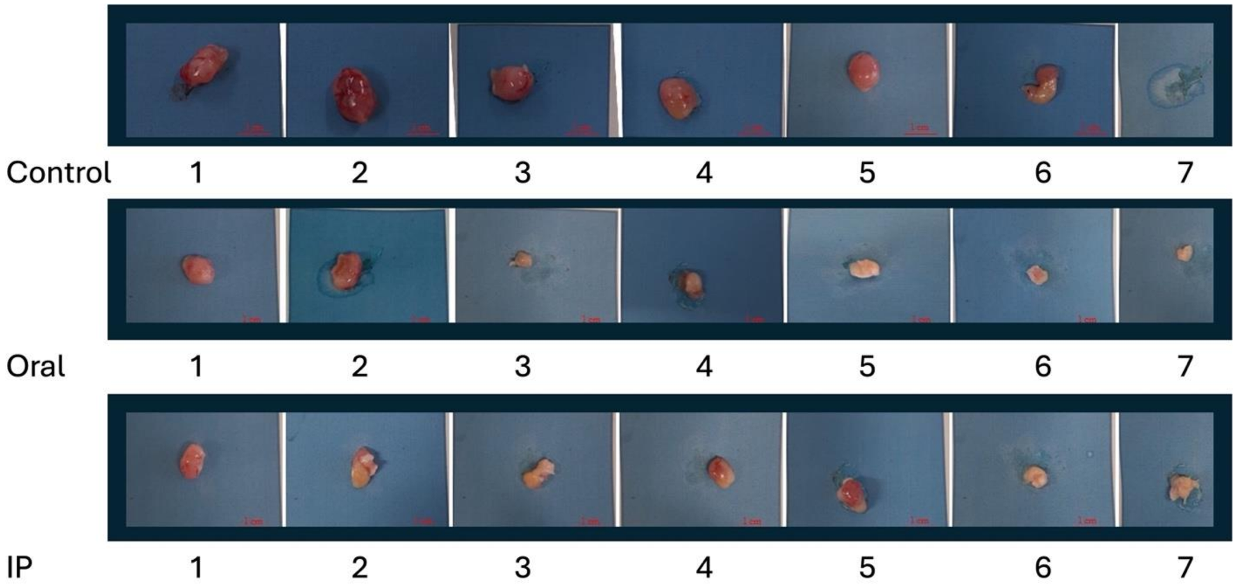

The tumours were removed after sacrifice and weighed for statistical comparison, which is shown in Figure 5 of the main text of the MS.

Table S1. List of functional annotations.

Category	Term	Count	%	PValue	Genes	Fold Enrichment	Benjamini	FDR
KEGG_PATHWAY	mmu04060:Cytokine-cytokine receptor interaction	11	91,66666667	1,17E-14	IL10, CCL25, CD40, CSF2, IL5, CCL5, IL1B, LEP, F	28,28795662	**9,36E-13**	7,14E-13
GOTERM_MF_DIRECT	GO:0005125~cytokine activity	9	75	9,20E-14	IL10, CCL25, CSF2, IL5, CCL5, IL1B, FASLG, CX3	59,36170213	**4,69E-12**	4,60E-12
GOTERM_BP_DIRECT	GO:0006954~inflammatory response	8	66,66666667	3,65E-10	CCL25, CD40, IL5, AXL, CCL5, IL1B, CX3CL1, IL1	33,44804655	**1,69E-07**	1,56E-07
GOTERM_BP_DIRECT	GO:0006955~immune response	8	66,66666667	2,36E-09	IL10, CCL25, CSF2, IL5, CCL5, IL1B, FASLG, CX3	25,59669211	**5,44E-07**	5,04E-07
GOTERM_BP_DIRECT	GO:0071347~cellular response to interleukin-1	5	41,66666667	8,28E-08	CCL25, CD40, CCL5, CX3CL1, IL17A	102,2306911	**1,27E-05**	1,18E-05
GOTERM_BP_DIRECT	GO:0042531~positive regulation of tyrosine phosphorylation of STAT protein	4	33,33333333	3,50E-06	CD40, CSF2, CCL5, LEP	117,6549708	**4,04E-04**	3,75E-04
GOTERM_BP_DIRECT	GO:0043491~protein kinase B signaling	4	33,33333333	5,21E-06	CD40, AXL, CCL5, IL1B	103,174359	**4,82E-04**	4,46E-04
GOTERM_BP_DIRECT	GO:0030593~neutrophil chemotaxis	4	33,33333333	1,05E-05	CCL25, CCL5, IL1B, CX3CL1	81,78455285	**8,10E-04**	7,50E-04
GOTERM_BP_DIRECT	GO:0008284~positive regulation of cell proliferation	6	50	1,24E-05	CSF2, IL5, IL1B, LEP, FASLG, CX3CL1	15,79199372	**8,15E-04**	7,55E-04
KEGG_PATHWAY	mmu04672:Intestinal immune network for IgA production	4	33,33333333	1,63E-05	IL10, CCL25, CD40, IL5	69,85271318	**6,51E-04**	4,96E-04
GOTERM_BP_DIRECT	GO:0071346~cellular response to interferon-gamma	4	33,33333333	2,54E-05	CCL25, CCL5, FASLG, CX3CL1	60,96666667	**0,001467734**	0,001359719
KEGG_PATHWAY	mmu05321:Inflammatory bowel disease	4	33,33333333	4,92E-05	IL10, IL5, IL1B, IL17A	48,44623656	**0,001032802**	7,88E-04
KEGG_PATHWAY	mmu05330:Allograft rejection	4	33,33333333	5,16E-05	IL10, CD40, IL5, FASLG	47,67724868	**0,001032802**	7,88E-04
GOTERM_BP_DIRECT	GO:0071356~cellular response to tumor necrosis factor	4	33,33333333	6,04E-05	CCL25, CD40, CCL5, CX3CL1	45,62131519	**0,003100878**	0,002872675
GOTERM_BP_DIRECT	GO:0043123~positive regulation of I-kappaB kinase/NF-kappaB signaling	4	33,33333333	6,80E-05	CD40, IL1B, FASLG, CX3CL1	43,83224401	**0,003143557**	0,002912213
GOTERM_BP_DIRECT	GO:0048245~eosinophil chemotaxis	3	25	8,77E-05	CCL25, CCL5, CX3CL1	193,4519231	**0,003683197**	0,003412139
KEGG_PATHWAY	mmu05320:Autoimmune thyroid disease	4	33,33333333	1,02E-04	IL10, CD40, IL5, FASLG	38,02109705	**0,001627847**	0,001241233
GOTERM_MF_DIRECT	GO:0048020~CCR chemokine receptor binding	3	25	1,03E-04	CCL25, CCL5, CX3CL1	178,8461538	**0,002614872**	0,0025636
GOTERM_BP_DIRECT	GO:0048247~lymphocyte chemotaxis	3	25	1,25E-04	CCL25, CCL5, CX3CL1	162,25	**0,004669553**	0,004325907
GOTERM_BP_DIRECT	GO:0043547~positive regulation of GTPase activity	4	33,33333333	1,31E-04	CCL25, CD40, CCL5, CX3CL1	35,11169284	**0,004669553**	0,004325907
KEGG_PATHWAY	mmu05323:Rheumatoid arthritis	4	33,33333333	1,36E-04	CSF2, CCL5, IL1B, IL17A	34,52490421	**0,001761849**	0,00134341
GOTERM_BP_DIRECT	GO:2001240~negative regulation of extrinsic apoptotic signaling pathway in absence o	3	25	1,51E-04	CSF2, IL1B, CX3CL1	147,9338235	**0,00498348**	0,00461673
KEGG_PATHWAY	mmu04657:IL-17 signaling pathway	4	33,33333333	1,65E-04	CSF2, IL5, IL1B, IL17A	32,29749104	**0,001761849**	0,00134341
KEGG_PATHWAY	mmu04061:Viral protein interaction with cytokine and cytokine receptor	4	33,33333333	1,76E-04	IL10, CCL25, CCL5, CX3CL1	31,61754386	**0,001761849**	0,00134341
GOTERM_BP_DIRECT	GO:0002548~monocyte chemotaxis	3	25	1,79E-04	CCL25, CCL5, CX3CL1	135,9391892	**0,005516863**	0,00511086
KEGG_PATHWAY	mmu05142:Chagas disease	4	33,33333333	2,24E-04	IL10, CCL5, IL1B, FASLG	29,1618123	**0,001990288**	0,001517594
GOTERM_BP_DIRECT	GO:0070374~positive regulation of ERK1 and ERK2 cascade	4	33,33333333	2,36E-04	CCL25, CCL5, IL1B, CX3CL1	28,78254649	**0,006516599**	0,006037023
GOTERM_BP_DIRECT	GO:0050796~regulation of insulin secretion	3	25	2,42E-04	CCL5, IL1B, LEP	116,9709302	**0,006516599**	0,006037023
GOTERM_BP_DIRECT	GO:0032735~positive regulation of interleukin-12 production	3	25	2,54E-04	CD40, LEP, IL17A	114,3125	**0,006516599**	0,006037023
KEGG_PATHWAY	mmu04668:TNF signaling pathway	4	33,33333333	2,94E-04	CSF2, CCL5, IL1B, CX3CL1	26,58112094	**0,002355657**	0,001796189
GOTERM_MF_DIRECT	GO:0008009~chemokine activity	3	25	2,97E-04	CCL25, CCL5, CX3CL1	105,6818182	**0,005044808**	0,00494589
GOTERM_BP_DIRECT	GO:0030335~positive regulation of cell migration	4	33,33333333	3,56E-04	CSF2, CCL5, IL1B, CX3CL1	25,02363184	**0,008520315**	0,007893279
GOTERM_BP_DIRECT	GO:0070098~chemokine-mediated signaling pathway	3	25	3,69E-04	CCL25, CCL5, CX3CL1	94,9009434	**0,008520315**	0,007893279
KEGG_PATHWAY	mmu05310:Asthma	3	25	4,00E-04	IL10, CD40, IL5	90,11	**0,002911111**	0,002219722
GOTERM_BP_DIRECT	GO:0046427~positive regulation of JAK-STAT cascade	3	25	4,73E-04	IL10, IL5, LEP	83,82916667	**0,010401203**	0,009635746
BIOCARTA	m_eosinophilsPathway:The Role of Eosinophils in the Chemokine Network of Allergy	3	25	5,02E-04	CSF2, IL5, CCL5	71,44444444	**0,015060336**	0,014056314
GOTERM_BP_DIRECT	GO:0032720~negative regulation of tumor necrosis factor production	3	25	6,80E-04	IL10, AXL, CX3CL1	69,85763889	**0,014286084**	0,013234727
GOTERM_BP_DIRECT	GO:0042102~positive regulation of T cell proliferation	3	25	8,39E-04	CCL5, IL1B, LEP	62,871875	**0,016853802**	0,015613479
KEGG_PATHWAY	mmu05143:African trypanosomiasis	3	25	9,80E-04	IL10, IL1B, FASLG	57,76282051	**0,006095315**	0,004647677
KEGG_PATHWAY	mmu04630:JAK-STAT signaling pathway	4	33,33333333	9,90E-04	IL10, CSF2, IL5, LEP	17,56530214	**0,006095315**	0,004647677
REACTOME_PATHWAY	R-MMU-380108~Chemokine receptors bind chemokines	3	25	0,001070456	CCL25, CCL5, CX3CL1	52,7654321	**0,052452355**	0,049240987
BIOCARTA	m_asbcellPathway:Antigen Dependent B Cell Activation	3	25	0,001487335	IL10, CD40, FASLG	42,86666667	**0,022310021**	0,020822686
GOTERM_BP_DIRECT	GO:0032755~positive regulation of interleukin-6 production	3	25	0,001606077	IL1B, LEP, IL17A	45,31306306	**0,030916984**	0,028641708
KEGG_PATHWAY	mmu05417:Lipid and atherosclerosis	4	33,33333333	0,001943993	CD40, CCL5, IL1B, FASLG	13,9058642	**0,011108534**	0,008470257
KEGG_PATHWAY	mmu05144:Malaria	3	25	0,002084561	IL10, CD40, IL1B	39,52192982	**0,011117657**	0,008477213
GOTERM_BP_DIRECT	GO:0051091~positive regulation of sequence-specific DNA binding transcription facto	3	25	0,002226766	IL10, IL5, IL1B	38,39503817	**0,040164665**	0,037208824
GOTERM_BP_DIRECT	GO:0051897~positive regulation of protein kinase B signaling	3	25	0,002260349	AXL, LEP, CX3CL1	38,10416667	**0,040164665**	0,037208824
GOTERM_BP_DIRECT	GO:0006935~chemotaxis	3	25	0,002397093	CCL25, CCL5, CX3CL1	36,98345588	**0,041016917**	0,037998356
GOTERM_BP_DIRECT	GO:0050999~regulation of nitric-oxide synthase activity	2	16,66666667	0,002731018	IL1B, LEP	670,6333333	**0,045061794**	0,041745558
KEGG_PATHWAY	mmu05163:Human cytomegalovirus infection	4	33,33333333	0,003157271	CCL5, IL1B, FASLG, CX3CL1	11,73307292	**0,015786353**	0,012037094
GOTERM_BP_DIRECT	GO:0019221~cytokine-mediated signaling pathway	3	25	0,003297769	IL5, IL1B, CX3CL1	31,4359375	**0,049757335**	0,04609554
GOTERM_BP_DIRECT	GO:0045766~positive regulation of angiogenesis	3	25	0,003297769	CD40, CCL5, IL1B	31,4359375	**0,049757335**	0,04609554
GOTERM_BP_DIRECT	GO:0045944~positive regulation of transcription from RNA polymerase II promoter	5	41,66666667	0,003338696	IL10, CD40, IL1B, CX3CL1, IL17A	6,760416667	**0,049757335**	0,04609554
GOTERM_MF_DIRECT	GO:0008083~growth factor activity	3	25	0,003432015	CSF2, IL5, LEP	30,79470199	**0,043758188**	0,042900184
GOTERM_BP_DIRECT	GO:0051092~positive regulation of NF-kappaB transcription factor activity	3	25	0,003502541	CD40, IL1B, CX3CL1	30,48333333	0,050567936	0,046846486
GOTERM_BP_DIRECT	GO:0031622~positive regulation of fever generation	2	16,66666667	0,003821525	CCL5, IL1B	479,0238095	0,051927782	0,048106257
GOTERM_BP_DIRECT	GO:0032747~positive regulation of interleukin-23 production	2	16,66666667	0,003821525	CSF2, IL17A	479,0238095	0,051927782	0,048106257
GOTERM_BP_DIRECT	GO:0043066~negative regulation of apoptotic process	4	33,33333333	0,004761285	IL10, AXL, LEP, CX3CL1	10,16111111	0,062848958	0,05822371
KEGG_PATHWAY	mmu04640:Hematopoietic cell lineage	3	25	0,005570035	CSF2, IL5, IL1B	23,96542553	0,026211928	0,019986595
GOTERM_BP_DIRECT	GO:0045906~negative regulation of vasoconstriction	2	16,66666667	0,005999288	LEP, CX3CL1	304,8333333	0,076990864	0,07132487
KEGG_PATHWAY	mmu04620:Toll-like receptor signaling pathway	3	25	0,006282703	CD40, CCL5, IL1B	22,5275	0,027923124	0,021291382
KEGG_PATHWAY	mmu04660:T cell receptor signaling pathway	3	25	0,00665397	IL10, CSF2, IL5	21,87135922	0,028016716	0,021362746
BIOCARTA	m_cytokinePathway:Cytokine Network	3	25	0,006706149	IL10, IL5, IL17A	20,41269841	0,067061494	0,062590727
GOTERM_BP_DIRECT	GO:0071803~positive regulation of podosome assembly	2	16,66666667	0,007086546	CSF2, IL5	257,9358974	0,088486058	0,081974097
KEGG_PATHWAY	mmu05146:Amoebiasis	3	25	0,007164336	IL10, CSF2, IL1B	21,05373832	0,028657343	0,021851224
GOTERM_MF_DIRECT	GO:0042379~chemokine receptor binding	2	16,66666667	0,007663414	CCL25, CCL5	238,4615385	0,078166824	0,076634141
GOTERM_BP_DIRECT	GO:0097028~dendritic cell differentiation	2	16,66666667	0,008715405	CSF2, AXL	209,5729167	0,103363888	0,095757022
GOTERM_BP_DIRECT	GO:0001934~positive regulation of protein phosphorylation	3	25	0,008725523	CD40, IL1B, LEP	19,05208333	0,103363888	0,095757022
BIOCARTA	m_inflamPathway:Cytokines and Inflammatory Response	3	25	0,010217283	IL10, CSF2, IL5	16,48717949	0,076629621	0,071520979
GOTERM_BP_DIRECT	GO:0071222~cellular response to lipopolysaccharide	3	25	0,011152226	IL10, AXL, IL1B	16,76583333	0,128737694	0,119263491
GOTERM_BP_DIRECT	GO:2000353~positive regulation of endothelial cell apoptotic process	2	16,66666667	0,011424774	CD40, FASLG	159,6746032	0,128737694	0,119263491
REACTOME_PATHWAY	R-MMU-375276~Peptide ligand-binding receptors	3	25	0,012096403	CCL25, CCL5, CX3CL1	15,31899642	0,296361874	0,278217269
KEGG_PATHWAY	mmu04936:Alcoholic liver disease	3	25	0,012189847	IL1B, FASLG, IL17A	15,97695035	0,046437513	0,035408604
GOTERM_BP_DIRECT	GO:0010759~positive regulation of macrophage chemotaxis	2	16,66666667	0,013587419	CCL5, CX3CL1	134,1266667	0,149461614	0,138462274
KEGG_PATHWAY	mmu04932:Non-alcoholic fatty liver disease	3	25	0,014783212	IL1B, LEP, FASLG	14,44070513	0,053757134	0,040989814
GOTERM_BP_DIRECT	GO:0002639~positive regulation of immunoglobulin production	2	16,66666667	0,016284678	IL10, IL5	111,7722222	0,172719465	0,160008509
GOTERM_BP_DIRECT	GO:1900745~positive regulation of p38MAPK cascade	2	16,66666667	0,016823325	IL1B, LEP	108,1666667	0,172719465	0,160008509
GOTERM_BP_DIRECT	GO:0050900~leukocyte migration	2	16,66666667	0,016823325	CCL25, IL1B	108,1666667	0,172719465	0,160008509
KEGG_PATHWAY	mmu05164:Influenza A	3	25	0,01798748	CCL5, IL1B, FASLG	13,0216763	0,062565149	0,047705926
GOTERM_BP_DIRECT	GO:0034341~response to interferon-gamma	2	16,66666667	0,018437655	CD40, CCL5	98,62254902	0,18123823	0,167900352
GOTERM_BP_DIRECT	GO:0045672~positive regulation of osteoclast differentiation	2	16,66666667	0,018437655	CCL5, IL17A	98,62254902	0,18123823	0,167900352
GOTERM_BP_DIRECT	GO:0010934~macrophage cytokine production	2	16,66666667	0,020049576	AXL, IL1B	90,62612613	0,192977173	0,17877539
GOTERM_BP_DIRECT	GO:0030168~platelet activation	2	16,66666667	0,021122853	AXL, CX3CL1	85,97863248	0,199158329	0,184501655
GOTERM_BP_DIRECT	GO:0042832~defense response to protozoan	2	16,66666667	0,021659091	IL10, CD40	83,82916667	0,200129996	0,185401815
KEGG_PATHWAY	mmu04062:Chemokine signaling pathway	3	25	0,021889495	CCL25, CCL5, CX3CL1	11,73307292	0,072964984	0,055635801
GOTERM_BP_DIRECT	GO:0001818~negative regulation of cytokine production	2	16,66666667	0,022195061	IL10, AXL	81,78455285	0,20106114	0,186264433
GOTERM_BP_DIRECT	GO:0032689~negative regulation of interferon-gamma production	2	16,66666667	0,022730764	IL10, AXL	79,83730159	0,201954099	0,187091676
GOTERM_BP_DIRECT	GO:0031663~lipopolysaccharide-mediated signaling pathway	2	16,66666667	0,023266201	CCL5, IL1B	77,98062016	0,202811036	0,187885548
GOTERM_MF_DIRECT	GO:0042056~chemoattractant activity	2	16,66666667	0,023409409	CCL5, CX3CL1	77,5	0,198979979	0,195078411
GOTERM_BP_DIRECT	GO:0010507~negative regulation of autophagy	2	16,66666667	0,024870911	IL10, LEP	72,89492754	0,212784463	0,197125
REACTOME_PATHWAY	R-MMU-418594~G alpha (i) signalling events	3	25	0,025227851	CCL25, CCL5, CX3CL1	10,39902676	0,303630907	0,285041259
GOTERM_BP_DIRECT	GO:0097192~extrinsic apoptotic signaling pathway in absence of ligand	2	16,66666667	0,025405282	IL1B, CX3CL1	71,34397163	0,213404367	0,197699284
GOTERM_BP_DIRECT	GO:0030890~positive regulation of B cell proliferation	2	16,66666667	0,026473224	CD40, IL5	68,43197279	0,218404101	0,202331072
REACTOME_PATHWAY	R-MMU-162582~Signal Transduction	6	50	0,028109661	CCL25, CSF2, AXL, CCL5, FASLG, CX3CL1	2,646849357	0,303630907	0,285041259
REACTOME_PATHWAY	R-MMU-373076~Class A/1 (Rhodopsin-like receptors)	3	25	0,031193551	CCL25, CCL5, CX3CL1	9,281216069	0,303630907	0,285041259
GOTERM_BP_DIRECT	GO:0014823~response to activity	2	16,66666667	0,031265813	IL10, LEP	57,81321839	0,253417643	0,23476786
GOTERM_BP_DIRECT	GO:0032757~positive regulation of interleukin-8 production	2	16,66666667	0,033919085	IL1B, LEP	53,22486772	0,270183054	0,250299453
GOTERM_BP_DIRECT	GO:0002376~immune system process	3	25	0,035077755	CD40, AXL, IL17A	9,111865942	0,27467666	0,25446236
BIOCARTA	m_bbcellPathway:Bystander B Cell Activation	2	16,66666667	0,036819879	CD40, FASLG	47,62962963	0,220919274	0,206191323
GOTERM_BP_DIRECT	GO:0045785~positive regulation of cell adhesion	2	16,66666667	0,038678331	CCL5, CX3CL1	46,57175926	0,296935873	0,275083449
GOTERM_BP_DIRECT	GO:0071407~cellular response to organic cyclic compound	2	16,66666667	0,039205819	CCL5, IL1B	45,93378995	0,296935873	0,275083449
GOTERM_BP_DIRECT	GO:0032868~response to insulin	2	16,66666667	0,041839311	IL10, LEP	42,98931624	0,308804472	0,286078602
GOTERM_BP_DIRECT	GO:0010628~positive regulation of gene expression	3	25	0,042109701	CSF2, CCL5, IL1B	8,245491803	0,308804472	0,286078602
GOTERM_BP_DIRECT	GO:0014068~positive regulation of phosphatidylinositol 3-kinase signaling	2	16,66666667	0,043941378	CCL5, LEP	40,89227642	0,312321791	0,289337071
GOTERM_BP_DIRECT	GO:0043406~positive regulation of MAP kinase activity	2	16,66666667	0,043941378	CD40, IL1B	40,89227642	0,312321791	0,289337071
GOTERM_BP_DIRECT	GO:0001819~positive regulation of cytokine production	2	16,66666667	0,045515176	IL10, IL1B	39,44901961	0,31279609	0,289776464
GOTERM_BP_DIRECT	GO:0043525~positive regulation of neuron apoptotic process	2	16,66666667	0,045515176	IL1B, FASLG	39,44901961	0,31279609	0,289776464
GOTERM_BP_DIRECT	GO:0048661~positive regulation of smooth muscle cell proliferation	2	16,66666667	0,046039251	CCL5, CX3CL1	38,99031008	0,31279609	0,289776464
REACTOME_PATHWAY	R-MMU-5218859~Regulated Necrosis	2	16,66666667	0,04676431	IL1B, FASLG	37,24618736	0,303630907	0,285041259
BIOCARTA	m_il5Pathway:IL 5 Signaling Pathway	2	16,66666667	0,048826684	IL5, IL1B	35,72222222	0,244133418	0,227857857
REACTOME_PATHWAY	R-MMU-500792~GPCR ligand binding	3	25	0,049344728	CCL25, CCL5, CX3CL1	7,231810491	0,303630907	0,285041259
REACTOME_PATHWAY	R-MMU-1280215~Cytokine Signaling in Immune system	3	25	0,049572393	CD40, CSF2, IL1B	7,21350211	0,303630907	0,285041259
GOTERM_BP_DIRECT	GO:0060326~cell chemotaxis	2	16,66666667	0,049700463	CCL25, CX3CL1	36,05555556	0,332777013	0,30828693
GOTERM_BP_DIRECT	GO:0050729~positive regulation of inflammatory response	2	16,66666667	0,053348896	IL1B, CX3CL1	33,53166667	0,35052825	0,324731799
GOTERM_BP_DIRECT	GO:0006909~phagocytosis	2	16,66666667	0,05386906	AXL, LEP	33,19966997	0,35052825	0,324731799
GOTERM_BP_DIRECT	GO:0071260~cellular response to mechanical stimulus	2	16,66666667	0,058538857	CD40, IL1B	30,48333333	0,371933345	0,344561627
GOTERM_BP_DIRECT	GO:0045893~positive regulation of transcription, DNA-templated	3	25	0,05934715	IL10, IL5, IL1B	6,815379404	0,371933345	0,344561627
GOTERM_BP_DIRECT	GO:0000165~MAPK cascade	2	16,66666667	0,059573739	CCL5, IL1B	29,9389881	0,371933345	0,344561627
GOTERM_BP_DIRECT	GO:0032760~positive regulation of tumor necrosis factor production	2	16,66666667	0,061124124	LEP, IL17A	29,15797101	0,376524607	0,348815004
GOTERM_BP_DIRECT	GO:0006874~cellular calcium ion homeostasis	2	16,66666667	0,062672186	CD40, CCL5	28,41666667	0,380980919	0,352943362
KEGG_PATHWAY	mmu05332:Graft-versus-host disease	2	16,66666667	0,074312999	IL1B, FASLG	23,83862434	0,226902413	0,17301309
KEGG_PATHWAY	mmu04623:Cytosolic DNA-sensing pathway	2	16,66666667	0,074312999	CCL5, IL1B	23,83862434	0,226902413	0,17301309
KEGG_PATHWAY	mmu04664:Fc epsilon RI signaling pathway	2	16,66666667	0,077723095	CSF2, IL5	22,75505051	0,226902413	0,17301309
KEGG_PATHWAY	mmu04940:Type I diabetes mellitus	2	16,66666667	0,082252125	IL1B, FASLG	21,4547619	0,226902413	0,17301309
KEGG_PATHWAY	mmu05140:Leishmaniasis	2	16,66666667	0,082252125	IL10, IL1B	21,4547619	0,226902413	0,17301309
BIOCARTA	m_stemPathway:Regulation of hematopoiesis by cytokines	2	16,66666667	0,084064991	CSF2, IL5	20,41269841	0,360278532	0,336259963
GOTERM_MF_DIRECT	GO:0005178~integrin binding	2	16,66666667	0,087419603	IL1B, CX3CL1	20,12987013	0,636914252	0,624425737
KEGG_PATHWAY	mmu05133:Pertussis	2	16,66666667	0,090129303	IL10, IL1B	19,504329	0,240344807	0,183262916
REACTOME_PATHWAY	R-MMU-388396~GPCR downstream signalling	3	25	0,094220828	CCL25, CCL5, CX3CL1	5,025279248	0,510454259	0,479201958
GOTERM_BP_DIRECT	GO:0032496~response to lipopolysaccharide	2	16,66666667	0,09614788	IL1B, FASLG	18,22373188	0,572445363	0,53031735
GOTERM_BP_DIRECT	GO:0050830~defense response to Gram-positive bacterium	2	16,66666667	0,09664662	IL1B, IL17A	18,12522523	0,572445363	0,53031735

A Gene Ontology (GO) and pathway-based analysis of the differentially expressed genes was performed using the Database for Annotation, Visualization and Integrated Discovery (DAVID) in order to assess the biological relevance of up/down-regulated genes of the DVA-treated tumours in comparison with the control ones.
